# Work-related posttraumatic stress disorder in paramedics in comparison to data from the general population of working age. A systematic review and meta-analysis

**DOI:** 10.3389/fpubh.2023.1151248

**Published:** 2023-03-09

**Authors:** Andreas Hoell, Eirini Kourmpeli, Harald Dressing

**Affiliations:** Department of Psychiatry and Psychotherapy, Central Institute of Mental Health, Medical Faculty Mannheim, University of Heidelberg, Mannheim, Germany

**Keywords:** paramedics, critical incidence, prevalence, meta-analysis, ambulance personnel, post-traumatic stress disorder

## Abstract

**Objective:**

Paramedics are at particularly high risk for developing posttraumatic stress disorders (PTSD). Hitherto, evidence for higher prevalence rates in paramedics compared to the general population is vague. We aimed to determine and compare 12-month prevalence of PTSD in paramedics and general population from high-income countries.

**Methods:**

We conducted systematic review processes to identify relevant studies for inclusion. For paramedics, we searched relevant databases, reference lists, and did citation tracking. Inclusion criteria were applied according to PICO. Quality of the studies was assessed using a validated methodological rating tool. Twelve-month prevalence data from all studies were pooled using random effects model. Subgroup analyses were performed to identify sources of heterogeneity.

**Results:**

In total, we found 41 distinct samples with 17,045 paramedics, 55 samples with 311,547 individuals from non-exposed general population, 39 samples with 118,806 individuals from populations affected by natural disasters, and 22 samples with 99,222 individuals from populations affected by human-made disasters. Pooled 12-month prevalence estimates of PTSD were 20.0, 3.1, 15.6, and 12.0%, respectively. Prevalence estimates in paramedics varied with methodological quality and measurement instrument. Paramedics reporting distinct critical incidences had lower pooled prevalence than paramedics reporting indistinct types of exposure.

**Conclusion:**

Paramedics have a pooled prevalence of PTSD that is considerably higher than rates of unexposed general population and populations affected by human-made disasters. Chronic exposure to low-threshold traumatic events during daily routine work is a risk factor for developing PTSD. Strategies to ensure long working lifetime are strongly needed.

## 1. Introduction

Paramedics encounter extreme experiences during their daily routines by providing physical and emotional support to afflicted individuals of critical incidents. Such stressful incidences include first aid for burned patients, accidents involving children, road traffic accidents, violent incidents (1, 2), and threats and acts of violence against paramedics themselves (3–5). Paramedics are repeatedly exposed to critical incidences and have longer and intimate contact with injured patients and/or relatives (6). Witnessing the suffering of others could interfere with the professional detached attitude (6). Experiences of occupation-related potentially traumatic incidences could possibly lead to posttraumatic stress disorders (PTSD).

However, type, frequency, and severity of exposure that need to be in causal relation to the diagnosis of PTSD differ between the current and former versions of the International Statistical Classification of Diseases and Related Health Problems (ICD) and the Diagnostic and Statistical Manual of Mental Disorders (DSM). For instance, the DSM-5 names the experience of one, more or the repeatedly exposure to traumatic events to oneself or being witness of such event(s) as fundamental for the diagnosis of PTSD, while the ICD-10 does not (7). However, the repeatedly exposure to critical incidences might have a cumulative effect which increases the risk for PTSD (8). The harmonization of exposure criteria is critical a) to correctly assess the occupational risk for PTSD in potentially high-risk groups, b) to adjust occupational stressors to the requirements of the daily routine of first responders (6), and c) to acknowledge the emotional burden of first responders leading to secondary traumatic stress (9). In addition to the exposure criteria, further peri-traumatic factors (like experiences of dissociation or acute stress reaction), and pre- and post-traumatic factors moderate or confound the relationship between intensity and frequency of critical incidents and the manifestation of PTSD (10). Some of these factors are related to operational and organizational workplace stressors (psychosocial work environment), demographic factors, personality (including coping behavior), and social network or perceived social support (10–14). They could serve as risk or protective factors for the development of PTSD in paramedics, but a recent review of longitudinal studies found inconclusive results (15).

There is evidence that paramedics develop worse physical and mental health conditions during their career than other employees with increased rates of mortality, occupational fatality, and early retirement (11, 14). They are at increased risk of developing PTSD owing to work-related traumatic events. Previous systematic reviews provided PTSD prevalence rates of 4.0–21.5% in paramedics (11, 14, 16–18). Contrary, the cross-national 12-month prevalence for PTSD from representative samples of 26 populations according to the World Mental Health Survey is considerably lower with 2.8% (19). Several methodological challenges, such as lack of a comparison group or comparisons with normative data limit the interpretation of aforementioned results. A recent meta-analysis that evaluated the relative occupational risk for PTSD in trauma-exposed groups compared to seemingly non trauma-exposed groups of employees was not able to calculate the relative occupational risk for PTSD in paramedics (20). To our knowledge, meta-analyses that compare the prevalence of PTSD in paramedics with normative data from national cohort studies or nationally representative samples are not available. In addition, there are only a few studies comparing the prevalence of PTSD in paramedics with samples from the general population that were exposed to major critical incidences like natural disasters, human-made disasters or terror attacks. Our study tries to bridge the gap. Furthermore, we believe that the risk to develop PTSD differs between the general population exposed to critical major incidents and paramedics. Peri-, pre-, and post-traumatic factors and the emotional involvement increase the vulnerability of paramedics for the development of PTSD.

We aimed to address the following research question: Do (P) paramedics have (I) due to work-related traumata (C) in comparison with the general working population (O) a higher prevalence of PTSD? We conducted a systematic review with meta-analysis to provide the 12-month prevalence of work-related PTSD in paramedics in high-income countries. We compared the pooled 12-monthe prevalence with 12-month prevalence of PTSD in (a.) Non-systematically trauma-exposed general working population and (b.) Trauma-exposed general population due to natural disasters and disasters caused by human beings (including terror attacks).

## 2. Methods

We adhered to the Preferred Reporting Items of Systematic Reviews and Meta-Analyses (PRISMA) statements (21) for study selection and reporting (Supplementary material 1). We formulated our research question and inclusion criteria according to the PICO-criteria. The study protocol was published a priori in PROSPERO (CRD42021273046).

### 2.1. Search strategy

We used a three-step search process to maximize the likelihood of obtaining all relevant published research on PTSD in paramedics. At first, we conducted a computerized literature search of eight international literature databases PubMed, CINAHL, PsycINFO, PSYNDEX, Academic Search Complete, Science Direct, Web of Science and PTSDpubs on 11 August 2021. We limited the literature search to publications from 01 January 1995 onwards due to the establishment and usage of DSM-IV and ICD-10 diagnostic manuals, but did not provide language restrictions as far as an abstract in English was available. We combined search terms like paramedics, emergency medical technicians or ambulance personnel with posttraumatic stress disorder or secondary traumatic stress. We searched abstracts, titles, key or text words, and medical subject headings (MeSH) including the above-mentioned terms, thesaurus terms, its abbreviations and different spellings. We adjusted the systematic search strings corresponding to the terminology of each database. Supplementary material 2 contains the complete search strings. We included observational studies only, like cohort studies, case-control studies, cross-sectional or longitudinal studies that had to be original research articles published in peer-reviewed academic journals. Secondly, we checked the reference lists of eligible records identified in step one and relevant systematic reviews (11, 14, 16, 17). Finally, we performed a Google Scholar citation-tracking search with the most relevant studies included during step 1 or 2 and the relevant systematic reviews cited above.

In order to identify comparable studies in the general working population, we used a three-step search process, too, but limited computerized literature search to PubMed. We conducted the three steps in December 2021 using the limiters mentioned above and considered only studies from high-income countries (HIC). We combined search terms like general population, national surveys or community sample with representative or random sample and HIC. Moreover, we added specific terms for natural disaster or disasters caused by human being to identify studies in trauma-exposed general populations. In terms of outcome, study design and language of publication, we applied the same search criteria as for paramedics (Search string provided in Supplementary material 3).

### 2.2. Selection process

Records of the computerized literature search for paramedics were transferred to Endnote X9.3 (Clarivate 120 Analytics, Philadelphia, Pennsylvania, USA). One author (EK) did the preliminary check for duplicates and types of publication. Two authors (AH & EK) screened all identified studies independently. First, they checked the titles and abstracts for eligibility and later the full-texts of identified potentially relevant articles. Additional articles identified through searches of reference lists and citation tracking were examined in full-text. In cases of disagreement, a third author (HD) was consulted to find a consensus through discussion. We repeated this process to identify the relevant studies for PTSD in the general populations.

### 2.3. Inclusion and exclusion criteria

#### 2.3.1. Population of interest (P)

Our target group “paramedics” was defined as follows: all individuals actively full-time employed during the survey period in pre-hospital emergency medical care and transport in HIC. These individuals had completed apprenticeships according to the country-specific regulations as (emergency) paramedics, emergency medical technicians (EMT), rescue assistant or ambulance drivers and were not classified as medical staff. We excluded individuals working in emergency departments, administrative ambulance personnel (like dispatchers), volunteers, students, and retired paramedics, but air, sea, or mountain rescue services, too. If studies reported on multiple occupational groups, we reported data referring to paramedics, if possible. If such mixed samples did not report outcomes on specific occupational groups, the proportion of paramedics of the total sample should comprise at least 50%.

We concentrated on studies from HIC to enlarge the homogeneity of job demands and additional work related stressors like working in war zones, or in areas of extreme impoverishment (14). We defined HIC according to the gross national income (GNI) threshold derived from the World Bank Group. HIC are economies with a GNI per capita above an annually alterable threshold that increased between 1995 and 2020 from $9,385 to $12,696. We included studies conducted in economies continuously classified as HIC for the past 10 years (2012–2021).

#### 2.3.2. Intervention (I)

We selected samples of paramedics that were exposed to work-related traumatic events either in form of discrete events or vague events due to daily routines. Discrete events were critical mass incidents or clearly recalled work related traumatizing events. Vague events were undistinguishable traumatic events experienced during regular duties with a cumulative, repetitive nature, or repeatedly witnessing the aftermath of a trauma experienced by another person (i.e. secondary traumatization).

#### 2.3.3. Comparison groups (C)

We were not able to identify any study in the current literature that provide comparisons on PTSD prevalence for paramedics and the general population using the same study design. Thus, we chose studies reporting on PTSD prevalence from the general population at employable age exclusively. We included three different kinds of studies: (1) general populations not systematically exposed to traumatic events, (2) general population exposed to human-made disasters, and (3) general population exposed to natural disasters. Systematically exposed populations were defined as those populations at employable age residing in designated exposure areas at the time of and up to 10 years after a large disaster (22). The comparison groups had to be representative or randomly drawn from the above-mentioned sampling frame. Studies with non-random or convenience samples were excluded.

#### 2.3.4. Outcome (O)

The outcomes of interest were PTSD, posttraumatic stress symptoms, or secondary traumatic stress related to diagnostic criteria according to DSM-IV, DSM-IV-TR, DSM-5, ICD-10, or ICD-11. Outcomes should be obtained via (diagnostic) interview or self-report with standardized validated assessments. We required the use of appropriate cut-off scores or validated diagnostic algorithms to determine PTSD. Symptoms should last at least 1 month. Outcomes had to be reported as point-prevalence or 12-month prevalence. We excluded studies reporting lifetime prevalence, and studies that assessed PTSD within the first 4 weeks after a critical incident. In case of different publications with data on the same sample, the most current one with the most comprehensive data was included.

### 2.4. Quality assessment

Two independent reviewers (AH and EK) examined the methodological quality of included studies. All discrepancies were resolved by discussion. We used the critical appraisal tool for cross-sectional studies (AXIS) to rate the methodological quality (23), because the majority of included studies was cross-sectional designed. The tool is easy to use, delivers comparable results, and was used in recent similar reviews (24). The AXIS tool assesses multiple sources of risk of bias (ROB) according to study design and methods (sampling frame, selection process, handling of non-response), quality of reporting (data description, consistency of reporting results, justification of conclusions), and ethical considerations (funding sources, conflicts of interest). The tool contains 20 questions; each positive answer graded one point. Thus, higher sum scores indicate lower ROB. We calculated relative quality ranks for included studies on paramedics by dividing each study score by the score of the highest scoring study. We theoretically achieved quality ranks from zero to one. The studies were classified according to their quality score into: high (>0.849 points), moderate (0.7–0.849 points), and low (<0.7 points) methodological quality. The assessment of the methodological quality was limited to studies conducted in paramedics (Supplementary material 4).

### 2.5. Data extraction

Data were extracted from included full-text articles by one author (EK) and a second author (AH) reviewed the extraction for accuracy including outcomes, general study characteristics and variables for subgroup analyses. Study characteristics (country, year of data collection, study design, sample size, sampling frame, and critical appraisal score), participant characteristics (mean age and percentage of included males), and trauma-related information (type of trauma exposure, PTSD measure, classification scheme, type of PTSD scale, and prevalence) were recorded using two predefined entry forms for paramedics and general population, respectively. We grouped countries according to continents (Europe, Asia and Oceania, America), and used three categories for years of data collection (1995–2005, 2006–2015, and 2016–2020). In case of unknown year of data collection, we defined year of data collection as 1 year prior to the year the article was accepted for publication, or in case of missing information on acceptance date, 2 years prior to the year of publication (17). We grouped information on PTSD measure (diagnosis, screening) and on type of PTSD scale (IES-(R): Impact of Event Scale (Revised), PDS: Posttraumatic Stress Diagnostic Scale, PCL: PTSD-Checklist and its different versions, PCL-5: PTSD Checklist for DSM-5, PSS: PTSD Symptom Scale, and other screeners like the Pittsburgh Sleep Quality Index-Addendum for PTSD, and the Primary Care PTSD Screen).

### 2.6. Statistical analysis

We performed a meta-analysis of proportions using the “metafor” package in R (25). We used random-effects meta-analytical models to calculated mean proportions and 95% confidence interval (95%CI) by weighting each individual study by its inverse variance (25). We performed Freeman-Tukey double arcsine transformation of proportions to stabilize variance (26). For the ease of interpretation, we reported back-transformed proportions.

We assessed heterogeneity between studies *via* Cochran's Q and quantified its magnitude using *I*^2^ and τ^2^ statistics. To identify outliers, we performed sensitivity analyses. We evaluated the influence of each included survey on the pooled prevalence estimate. In order to determine the impact of potential publication biases, funnel plots were visually inspected, the trim and fill method to estimate potentially missing studies due to extreme results on one side of the funnel plot was used (27), and Egger's tests for funnel plot-asymmetry were applied (28). Statistical significance was set at *p* < *0.05*.

We used a series of subgroup analyses for studies in paramedics to evaluate the potential influence of categorical variables. Predictors of heterogeneity were exposure type, continent, year of data collection, type of PTSD scale, and methodological quality. Whereas, just one study used a clinical diagnosis to verify PTSD in paramedics, several studies used some version of the IES. The IES might have the tendency to over-report prevalence of PTSD, because it does not access the symptom cluster of hyper-arousal (29). In order to quantify the influence of the IES, we performed PTSD-scale adjusted subgroup analysis for paramedics and the general populations.

We compared paramedics with general populations in additional subgroup analyses. All subgroup analyses were conducted with mixed-effects models, whereat random-effects models determine subgroup-specific effects considering within-group variances, and fixed-effects models test between subgroup-specific effects. Results were presented using transformed values.

## 3. Results

### 3.1. Review of selected studies

**Figure 1:**
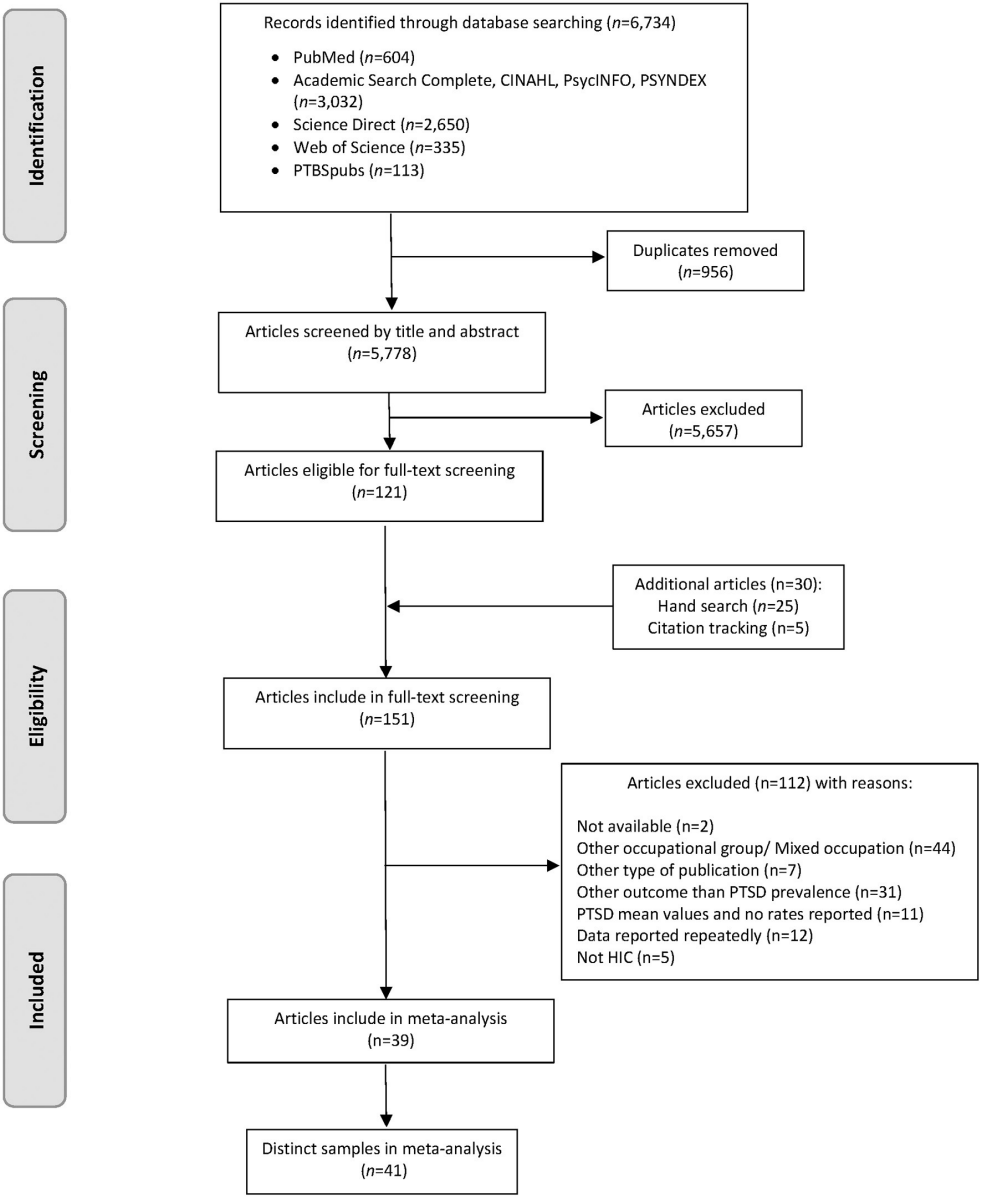
Flow diagram of the screening and study selection process of paramedics. PTSD, Posttraumatic Stress Disorder; HIC, High Income Country.

**Figure 2:**
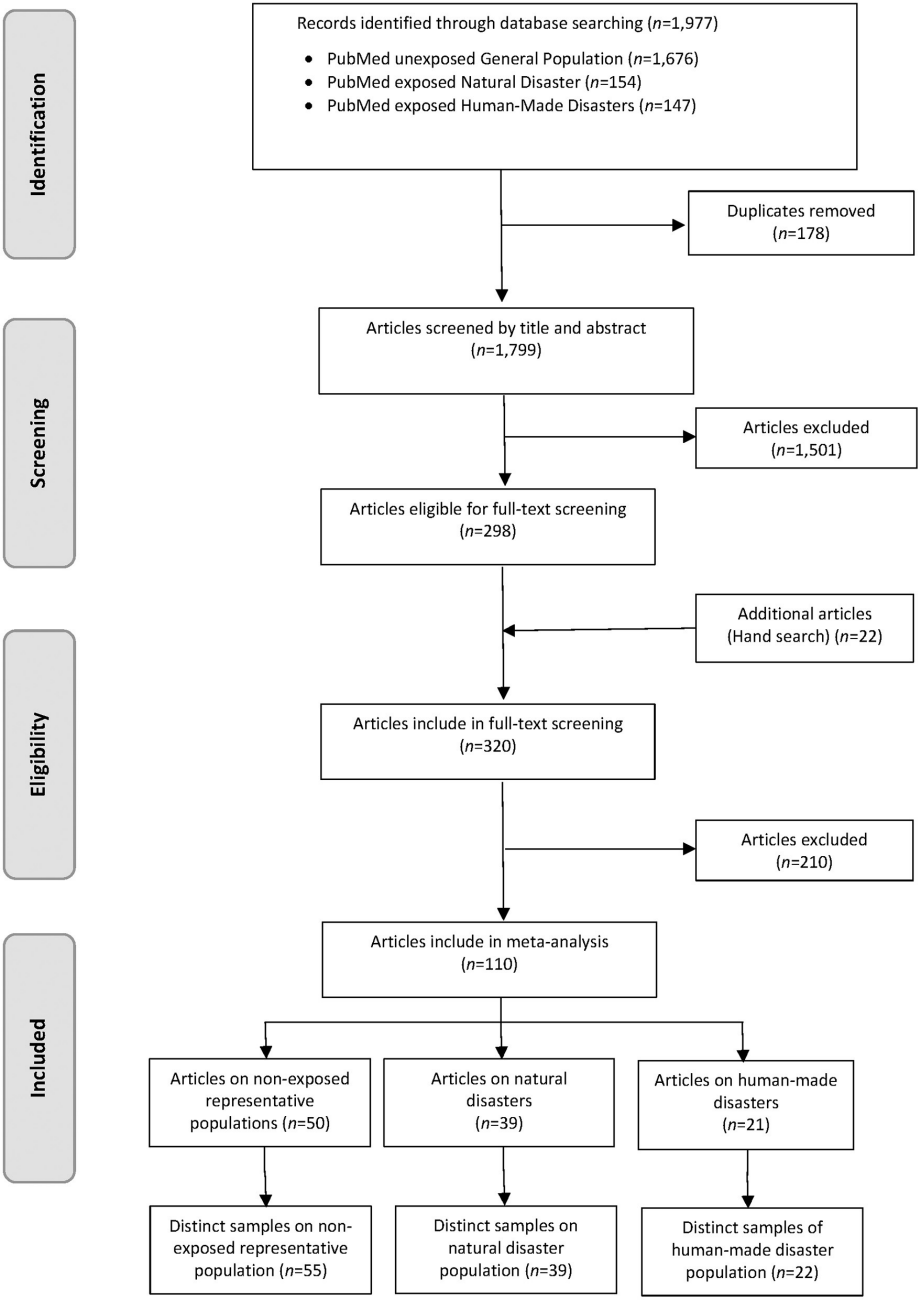
Flow diagram of the screening and study selection process of general populations of employable age.

Regarding studies on PTSD among paramedics, our search strategy totaled 6,734 records, with 955 duplicates. We excluded another 5,657 articles after reading titles and abstracts. We selected 121 articles for full-text review. The hand search of reference lists and the citation tracking provided 30 additional records. In total 39 full-text articles, describing 41 mutually exclusive studies on PTSD among paramedics, met the inclusion criteria for our meta-analysis (1–4, 30–64) (Figure 1). There was a substantial agreement between raters during the selection process with *Cohens-*κ of 85% (65). The systematic literature search for the general population at employable age provided 1,977 records including 178 duplicates. Figure 2 shows the complete selection process. In total 110 articles met the inclusion criteria. Of these, 50 articles reported on PTSD in unexposed general populations, 39 in exposed populations due to natural disasters and 21 in exposed populations due to human-made disasters. Characteristics of included studies are presented in Supplementary materials 5, 6.

### 3.2. 12-month prevalence of PTSD among paramedics

Data on prevalence of PTSD among paramedics could be retrieved from *k* = 41 surveys including n = 17,045 paramedics. The average age of participants was 36.9 years (*SD* = 3.3, *k* = 30). The majority of participants were male (69.7%, *k* = 37). In total, 19.5% of the included studies consisted of mixed samples with the majority being paramedics. The studies originated from 18 countries, with the highest number from the USA (*k* = 8). One survey used a diagnostic instrument. All other studies used screening tools with specific cut-offs. More than half of the studies (51.2%) used symptom terminology according to DSM-IV to assess PTSD prevalence, 17.1% used DSM-5 criteria and 31.7% no specific classification scheme. Most studies were done in emergency medical services (EMS) located solely in large urban areas (51.2%), 7.3% solely in rural area, and 36.6% in EMS from both areas. Another 4.9% did not specify locations of EMS. Regarding the methodological quality, two studies scored 17 out of 20 points and we assigned a rank of one. According to relative quality ranks, we classified 29.3% of studies as high quality, 39.0% as medium quality and 31.7% as low quality studies (Supplementary material 4).

**Figure 3:**
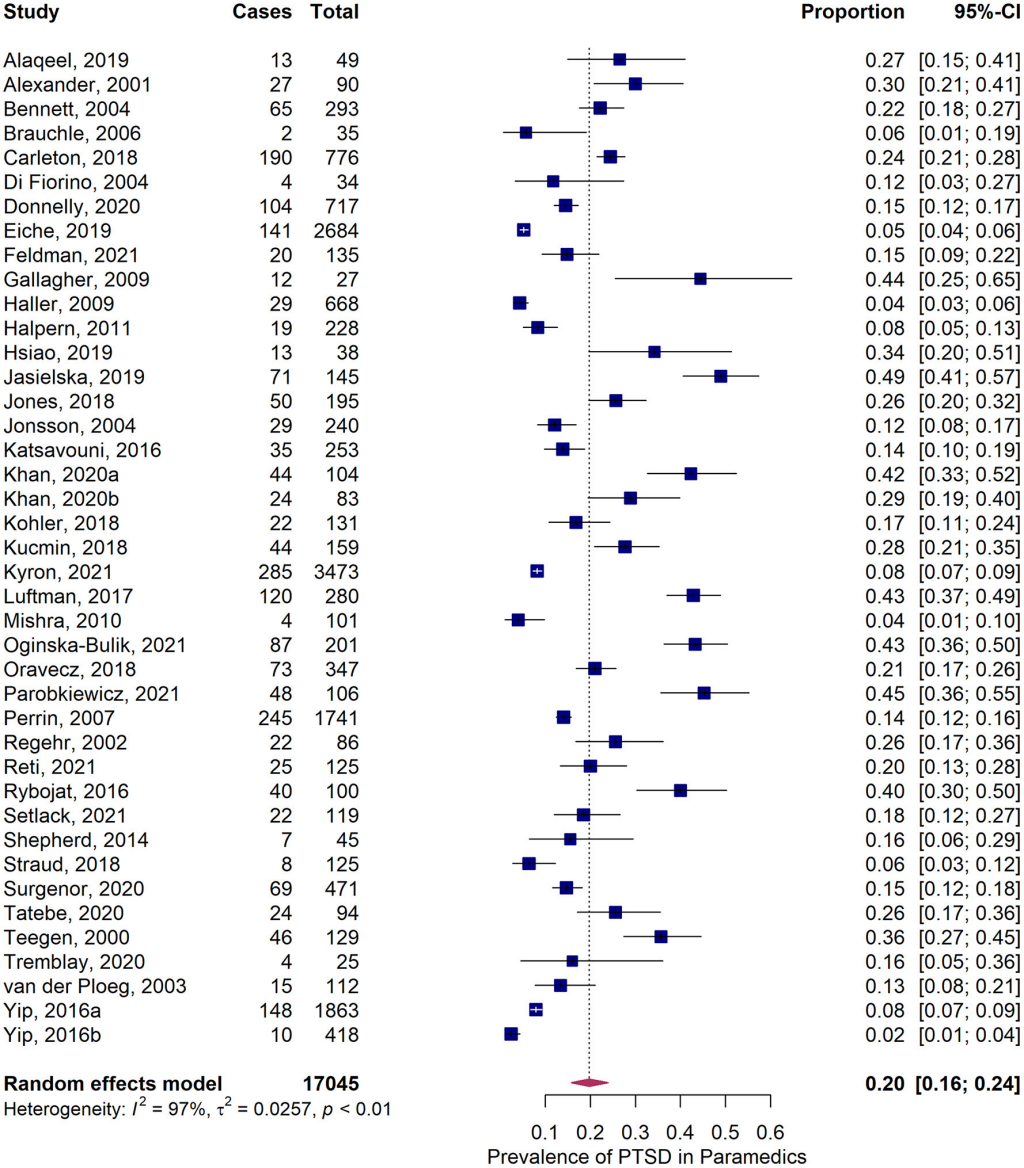
Forest plot of 12-month prevalence of posttraumatic stress disorder (PTSD) in paramedics.

The pooled 12-month prevalence estimate of PTSD in paramedics was 20.0% (*95%CI* = 16.1–24.3%) (Figure 3). We found significant heterogeneity among included studies, *Q* = 1,243.9, *p* < 0.001, *I*^2^ = 97.5%, and τ^2^ = 0.026, but could not identify any significant outlier. The funnel plot revealed slight asymmetry (Supplementary material 7) and the Egger's test confirmed a right-skewed publication bias (*p* = 0.039), although the trim and fill method indicated that there was no (potentially) missing study on the left side of the funnel plot.

The results of subgroup analyses of methodological and contextual variables on pooled prevalence estimates are depicted in Table 1. Studies with low methodological quality reported significantly higher prevalence estimates than studies with high quality (transformed estimate = 0.15, *p* = 0.038). Type of exposure influenced the prevalence estimate significantly: Paramedics exposed to vague events had significantly higher prevalence estimates (23.2%) compared to paramedics exposed to discrete events (15.2%, transformed estimate = 0.10, χ^2^ = 4.29, *df* = 1, p = 0.038). Surveys conducted with the IES revealed partly notable higher prevalence estimates compared to surveys using other screening-tools, especially the PDS. Overall, estimates were largely comparable (χ^2^ = 5.51, *df* = 4, *p* = 0.24; Supplementary material 8). Subgroup analyses according to continents and years of data collection did not provide significantly different prevalence estimates.

**Table 1:** Estimated pooled prevalence rates of PTSD in paramedics by subgroup variables.

**Subgroup variables**	**k**	**N**	**Estimate**	**SE**	**95%CI**	***p*-value**
**Exposure type**						
Discrete events	17	6,822	(reference)			
Vague events	24	10,223	0.10	0.05	0.01 – 0.20	**0.038**
**Critical appraisal**						
High score	12	12,173	(reference)			
Medium score	16	2,736	0.05	0.06	−0.07 – 0.17	0.414
Low score	13	2,136	0.15	0.07	0.01 – 0.29	**0.038**
**PTSD screening** ^a^						
IES	10	1,397	(reference)			
PDS	4	1,041	−0.18	0.09	−0.36 – 0.00	**0.037**
PCL-5	7	5,259	−0.05	0.08	−0.20 – 0.09	0.478
PCL (other versions)	14	5,910	−0.11	0.07	−0.24 – 0.03	0.125
All other screeners	5	3,404	−0.01	0.11	−0.23 – 0.20	0.911
**Year of data collection**						
Up to 2005	9	2,760	(reference)			
2006–2015	11	4,330	−0.04	0.07	−0.19 – 0.11	0.588
2016–2020	21	9,955	0.05	0.05	−0.05 – 0.15	0.339
**Continent**						
Europe	19	5,799	(reference)			
Asia, Oceania	7	4,343	0.01	0.07	−0.13 – 0.15	0.874
North America	15	6,903	−0.09	0.06	−0.20 – 0.02	0.115

a One study with diagnosis left out. PTSD, posttraumatic stress disorder; SE, standard error; 95%CI, 95% confidence interval; IES, Impact of Event Scale; PDS, Posttraumatic Diagnostic Scale; PCL-5, Posttraumatic Stress Disorder Checklist for DSM-5; PCL, other versions of the Posttraumatic Stress Disorder Checklist. The bold values indicate *p* < 0.05.

### 3.3. 12-month prevalence of PTSD in comparison groups

Data on prevalence of PTSD in non-systematically trauma-exposed general population could be retrieved from *k* = 55 surveys including n = 311,547 individuals. The majority of surveys were conducted in Europe (60%), 20% in Asia and Oceania, and 20% in North America. The majority of surveys with a share of 71% of all participants used clinical diagnostic interviews to detect prevalence of PTSD. We were able to select 39 surveys with n = 118,806 individuals exposed to natural disasters. The surveys were conducted to nearly equal proportions in North America (36%), in Europe (33%), and Asia/ Oceania (28%). Just one survey was conducted in South America (3%). Clinical diagnostics was used in 10.3% of included surveys. Finally, we identified *k* = 22 surveys with *n* = 99,222 individuals exposed to human-made disasters. The majority of surveys were conducted in North America (50%), followed by Europe (27%) and Asia/ Oceania (23%). Clinical diagnostics was used in 9.1% of included surveys.

Pooled 12-month prevalence of PTSD were 3.06% (*95%CI* = 2.31%-3.90%) in non-systematically trauma-exposed individuals, 15.59% (*95%CI* = 11.93%-19.63%) in individuals exposed to natural disasters, and 12.02% (*95%CI* = 9.17%-15.20%) in individuals exposed to human-made disasters. We found significant heterogeneity within all comparison groups (Supplementary materials 9–11). Funnel plots and tests of funnel plot asymmetry did not indicate publication biases (Supplementary materials 12–14).

We compared pooled 12-month prevalence of PTSD in paramedics with pooled 12-month prevalence of PTSD of each comparison group and found significantly larger double-arcsine transformed estimates in paramedics compared to non-systematically trauma-exposed general population (0.29, *95%CI* = 0.23–0.34, p < 0.001), and compared to populations exposed to human-made disasters (0.11, *95%CI* = *0.04–0.18*, p = 0.002) (Table 2). Estimates did not differ significantly between paramedics and populations exposed to natural disasters. Subgroup analyses in general populations revealed that surveys using screening tools had significantly higher pooled PTSD estimates than surveys using diagnostic interviews, and surveys conducted with the IES scale provided higher prevalence rates than any other screening tool (data not shown).

**Table 2:** Pooled prevalence estimates of PTSD in paramedics compared to unexposed and exposed groups of general population to major disasters.

**Subgroup variables**	***k***	***N***	**Transformed estimate**	**SE**	**95%CI**	***p*-value**
GP unexposed	55	311,547	(reference)			
Paramedics	41	17,045	0.29	0.03	0.23 – 0.34	**< 0.001**
GP exposed to ND	39	118,806	(reference)			
Paramedics	41	17,045	0.06	0.04	−0.02 – 0.13	0.123
GP exposed to HMD	22	99,222	(reference)			
Paramedics	41	17,045	0.11	0.04	0.04 – 0.18	**0.002**

PTSD, posttraumatic stress disorder; SE, standard error; 95%CI, 95% confidence interval; GP, general population; ND, natural disaster; HMD, human-made disaster. The bold values indicate *p* < 0.05.

## 4. Discussion

We provided the largest and most comprehensive exploration of PTSD in paramedics published to date with more than 17,000 individuals from HIC. We found large pooled work-related 12-month PTSD prevalence in paramedics with 20%. This estimate is larger than previously reported in meta-analysis on ambulance personnel with pooled estimates ranging from 11% to 14.6% (16, 17), although other reviews suggest that prevalence rates could be around 20% building upon prevalence rates from single studies (11, 14). The variation in pooled prevalence estimates could arise from different inclusion criteria. For instance, Berger et al. excluded studies using the IES, because the scale did not cover the DSM-IV core criteria of hyper-arousal (16), which might lead to overinflated prevalence estimates of PTSD (29). We included four studies using the IES and six studies using the revised version of the IES, which included all three symptom clusters according to DSM-IV: intrusion, avoidance and hyper-arousal. Four of the eighth studies on paramedics included in Berger et al. were also included in a meta-analysis on common mental disorders in ambulance personnel by Petrie and colleagues (17). Their meta-analysis included 13 studies on PTSD prevalence in ambulance personnel with nearly 2,800 individuals. Nevertheless, the total sample contained student paramedics, dispatcher, and administrative staff leading to possibly lower prevalence estimates.

One of five paramedics report symptoms of PTSD. It is imperative to continue efforts to implement mental health initiatives for ambulance personnel. These include standardized pre-employment selection processes, on-the-job training or education concerning mental health and well-being, and social embeddedness (66, 67). A further target should be the experienced emotional burden in conjunction with the physical and mental support of affected persons, because the lack of distancing might be responsible that paramedics were more affected with PTSD symptoms than any other group of professional first responders (16). We have to admit that the assumption of a unidirectional linear relationship between PTSD symptomatology and the severity and nature of critical incidences, is an oversimplification of underlying complex interactions of pre-, peri-, and post-disaster factors (10), because intra-individual differences in vulnerability, resilience and personality traits might be important moderator of this relationship (13). In addition, non-randomized cross-sectional studies with retrospective queries of traumatizing events and PTSD symptoms do not permit reliable statements on causality. We could not identify any study that directly compared PTSD prevalence between paramedics and GP. Thus, we used indirect comparisons with pooled data extracted from three systematic reviews of studies on PTSD in GP from HIC. We found considerable higher prevalence rates of probable PTSD in paramedics (20%) compared to non-systematically trauma-exposed GP (3%) and compared to GP experiencing human-made disasters (12%). Prevalence rates of probable PTSD differed marginally compared to GP experiencing natural disasters (16%). We were able to substantiate the frequently reported large difference in prevalence rates between paramedics and non-systematically trauma-exposed GP. In total, we could show that GP exposed to critical major incidents bear a lower risk to develop PTSD than paramedics do. We assume that type and severity of trauma is jointly responsible for this effect. Likewise, the literature suggests a relationship between peri-disaster factors and PTSD (18), but inconsistencies in definition and operationalization of peri-disaster factors inhibit the examination of their contribution to PTSD in our analyses. Some included studies in our analyses did not provide data on severity of single critical incidents, but on chronic exposure to (low-threshold) traumatic events or on concepts known as secondary traumatization. Thus, we compared the influence of chronic exposure to (low-threshold) traumatic events due to daily routines with the influence of reported discrete critical incidents on probable PTSD. Many studies used unexposed individuals of a specific occupational group to a large-scale disaster to prove the concept that devastating events are related to the development of PTSD. For example, Witteveen et al. found that fire fighters and police officers exposed to a large-scale human-made disaster showed higher rates of PTSD prevalence than non-exposed controls (68). In opposition to such studies, we found higher work-related probable PTSD in paramedics who could not relate their symptomatology of PTSD to discrete but cumulative incidents. Our findings coincide with findings from other systematic reviews that stated that chronic exposure to traumatic events is at least equally responsible for the development of PTSD like large-scale traumatic incidents (8, 16). Consequently, cumulative or chronic exposure to low-threshold traumatic events during daily routine work might cause work-related PTSD. Current neurobiological research refers to the importance of chronic stress and fear conditioning for the aetiopathogenesis of PTSD, i.e., the hippocampal sensitization due to chronic stress (8, 69). In relation to the aforementioned productivity issues and microeconomic factors, it is of utmost importance to develop strategies to ensure long working lifetime (6, 13).

At the same time, the following must be considered: We investigated work-related PTSD in active paramedics only. Thus, we probably underestimated the true prevalence, because we did not consider retired paramedics, paramedics on sick leave, and currently unemployed paramedics who might demonstrated higher rates on PTSD. Concomitantly, the healthy worker effect might systematically bias results. Self-selection or employment-selection provided a group of employees typically characterized by lower morbidity that might underestimated negative work-related impacts on mental health (6). Paramedics is a male dominated occupation. Nearly 70% of the workforce in included studies was of male gender. Studies on mental health of GP indicate that the prevalence of PTSD is higher in female gender (70, 71). However, our findings have to be interpreted with caution, because we were not able to provide any statistical adjustments for the different populations by taking into account gender, age, or employment rate (72). Consistent data were not available.

Beside subgroup analysis on trauma type, we performed additional subgroup analyses to understand the nature of substantial heterogeneity found in our meta-analysis on paramedics. We found that prevalence estimates of probable PTSD were biased by study quality, and concomitantly sample size. Studies with higher methodological quality (and larger sample sizes) provided on average lower prevalence estimates than studies with lower methodological quality. Wagner and colleagues found the same effect (18). Nevertheless, we could not find a relation between PTSD prevalence estimates and year of publication. Petrie et al. found a decrease of prevalence rates over time and explained this finding with (1) an increase in underreporting of PTSD due to stigma or fear of organizational consequences, and (2) an increase in awareness of mental health issues in ambulance or rescue stations (17). We like to add to these apparently contradicting arguments that there is generally weak evidence for work-place interventions in paramedics (67, 73). Especially, post-incidence support processes show contradictory results (67).

In addition, we found that PTSD prevalence rates were equal across included continents, probably because of including HIC only. Contrary, Berger et al. found higher prevalence estimates in rescue workers from Asia as compared to Europe (16). However, these samples were mostly of mixed occupations from developing countries. Lastly, there is an insufficient number of studies using diagnostic interviews to estimate prevalence of PTSD. Almost all studies used screening instruments, and we proved that the IES-(R) has the tendency to provide higher PTSD rates in paramedics than all other screenings, especially the PDS. The PDS was developed in accordance with the diagnostic criteria of DSM-IV and showed high diagnostic agreement with structured clinical interviews (74). Nevertheless, we were not able to explain the massive heterogeneity. We could not synthesize and control for other inter-individual factors like coping behavior, personality or sociodemographic factors due to missing information (18).

A number of limitations have to be considered regarding our review and the included studies. We treated all three categories of studies concerning GP like subgroups and compared each other with paramedics. It was the only way to achieve confidence limits, but comparability is inter alia limited due to different study designs, methods, and years of data collection. No computation of relative risk was possible. The existence of latent variables explaining differences between groups has to be acknowledged. Furthermore, we were confronted with publication bias in studies on paramedics and a generally large and unexplainable heterogeneity. Due to different objectives and methods, important information on study characteristics could not be synthesized. In case of studies in GP exposed or unexposed to major critical incidences, we did not test causes for huge heterogeneity, although we admit that types and consequences of exposure varied considerably. Many studies used self-reporting measures instead of diagnostic assessment to assess symptoms of PTSD and measures varied widely across studies. Self-report measures are not equivalent to clinician's diagnoses. Thus, important issues are raised concerning comparability and overestimation within data (75). Furthermore, retrospective self-reports could produce false or exaggerated psychological symptoms. Artifactual covariance could occur while the same person assesses the predictor (critical incident) as well as the criterion (probable PTSD) (76). Most studies in paramedics were cross-sectional in design questioning the causal implication. Hence, the reason for high PTSD prevalence estimates in paramedics remains unknown and directions between causes and effects are ambiguous. Although included studies in GP were largely representative for the target populations, studies in paramedics might be not. We cannot rule out self-selection of participants into the studies, because response rates were unknown or low. The term paramedic does not follow a uniform definition. Definitions varied between included studies. We included EMTs and ambulance drivers as well. In some cases (*k* = 3) we included mixed samples. Although we left out managerial staff and dispatchers, the type of work of paramedics, and differences in training level was not specified. In total, the results should be viewed with caution. The heterogeneity between studies in addition to potential measurement bias due to different PTSD measurements make it difficult to draw definitive conclusions about the prevalence of PTSD in paramedics. The focus on HIC may limit the generalizability of the findings to populations of countries classified as low- or middle-income countries. It is quite conceivable that the prevalence of other mental disorders, e.g., depression, is also increased in the group of paramedics. However, other mental disorders were not the subject of this meta-analysis. Studies regarding other mental disorders are certainly interesting, but require further meta-analytical methods, which could not be done in this context.

The basic strengths of our review are its comprehensive and rigorous study selection and its appropriate use of a quality assessment. Concerning future work on PTSD in paramedics, there is a need for prospective studies with case control designs, providing large samples, rigorous methods, and a combination of clinical interview and established screening instrument.

## Data availability statement

The data analyzed in this study is subject to the following licenses/restrictions: The original contributions presented in this study can be obtained upon request from the corresponding author. Requests to access these datasets should be directed to andreas.hoell@zi-mannheim.de.

## Author contributions

HD and AH had the idea for the manuscript. EK and AH did the literature search. EK did the data analysis. AH wrote the first draft of the manuscript and all authors commented on previous versions of the manuscript. All authors critically reviewed the included studies, contributed to the study conception and design, and read and approved the final version.
