# Supplementary material for: Work-related posttraumatic stress disorder in paramedics in comparison to data from the general population of working age. A systematic review and meta-analysis
Source: Front Public Health. 2023 Mar 9;11:1151248. doi: 10.3389/fpubh.2023.1151248 (PMC10035789; doi:10.3389/fpubh.2023.1151248)
Supplement: Supplementary file 1 [file Data_Sheet_1.ZIP › S2 Search-Strategy_PTSD_Paramedics.pdf]

Search strings for systematic review on risk for post-traumatic stress disorder among paramedics

Table 1: PubMed/ Medline

1	Emergency Medical Technicians (MeSH Terms)
2	Emergency Medic* Technician* (Text Word)
3	Paramedic* (Text Word)
4	Emergency Paramedic* (Text Word)
5	Paramedic* Personnel (Text Word)
6	Ambulance personnel (Text Word)
7	Or/1-6
8	Mental Health (MeSH Terms)
9	Mental Disorders (MeSH Terms)
10	Mental Disorder* (Text Word)
11	Mental Illness* (Text Word)
12	Stress Disorders, Post-Traumatic (MeSH Terms)
13	Post?traumatic Stress Disorder* (Text Word)
14	Posttraumatic Stress Disorder* (Text Word)
15	Post?traumatic stress (Text Word)
16	PTSD* (Text Word)
17	Stress disorder* (Text Word)
18	Or/8-17
19	7 And 18
20	Limit 19 –Filter: Publication date from 1994/01/01 to 2021/12/31

Table 2: Ebsco – including Academic Search Complete, CINAHL, PsycINFO, PSYNDEX

S1	Emergency medic* technician* [Search Modes: Find all my search terms]
S2	Emergency Paramedic* [Search Modes: Find all my search terms]
S3	Paramedic*[Search Modes: Find all my search terms]
S4	Paramedic* Personnel [Search Modes: Find all my search terms]
S5	Ambulance personnel [Search Modes: Find all my search terms]
S6	S1 OR S2 OR S3 OR S4 OR S5
S7	Mental health OR Mental disorder* OR Mental Illness* [Search Modes: Find all my search terms]
S8	Stress disorders, post-traumatic [Search Modes: Find all my search terms]
S9	Stress
S10	Posttraumatic stress disorder OR Post traumatic stress disorder OR Post-traumatic stress disorder OR PTSD [Search Modes: Find all my search terms]
S11	S7 OR S8 OR S9 OR S10
S12	S6 AND S11
S13	Limit S12 – Limiters: Published date: 19940101-20211231; Scholarly (Peer Reviewed) Journals

Table 3: Science Direct

1	“Emergency Medical Technicians” [find this term]
2	Paramedics [find this term]
3	“Ambulance personnel” [find this term]
4	1 OR 2 OR 3
5	“Mental Health”
6	“Mental Disorder”
7	Post Traumatic
8	Posttraumatic
9	5 OR 6 OR 7 OR 8
10	4 AND 9
11	Limit 10 – Refined by years: 1994-2021; Article Type: Review articles AND Research articles

Table 4: Web of Science/Social Sciences Citation Index (SSCI)

#1	TS= Emergency Medic* Technician*
#2	TS= Paramedic*
#3	TS= Ambulance personnel
#4	ALL= Emergency Medic* Technician*
#5	ALL= Emergency Paramedic* OR Paramedic* Personnel OR Ambulance personnel
#6	All= Paramedic*
#7	#1 OR #2 OR #3 OR #4 OR #5 OR #6
#8	TS= Mental Health
#9	TS= Mental Disorder*
#10	TS= Mental Illness*
#11	TS= Post-traumatic Stress* OR Posttraumatic Stress* OR Post Traumatic Stress*
#12	ALL= PTSD OR Post-traumatic Stress* OR Posttraumatic Stress* OR Post Traumatic Stress*
#13	#8 OR #9 OR #10 OR #11 OR #12
#14	#7 AND #13
#15	Limits #14 – Indexes=SSCI Timespan 1994-2021

Table 5: PTSDpubs (Electronic Index to the Traumatic Stress Literature, formerly known as PILOTS)

S1	Emergency Medic* Technician*
S2	Paramedic*
S3	Ambulance personnel
S4	Emergency Paramedic*
S5	Paramedic* Personnel
S6	S1 OR S2 OR S3 OR S4 OR S5
S7	Post-traumatic Stress* OR Posttraumatic Stress* OR Post Traumatic Stress*
S8	PTSD
S9	S7 OR S8
S10	S6 AND S9
S11	Limits S10 – Publication year: 1994-2021; Document type: Journal Article
