# Supplementary material for: Work-related posttraumatic stress disorder in paramedics in comparison to data from the general population of working age. A systematic review and meta-analysis
Source: Front Public Health. 2023 Mar 9;11:1151248. doi: 10.3389/fpubh.2023.1151248 (PMC10035789; doi:10.3389/fpubh.2023.1151248)
Supplement: Supplementary file 1 [file Data_Sheet_1.ZIP › S3 Search-Strategy_PTSD_OECD_High-income_Countries.docx]

**Search strings for post-traumatic stress disorder (PTSD) in the general population OR representative samples of OECD High-income countries**

**PubMed/ Medline**

**1. Syndrome [Title/Abstract]**

**(((post-traumatic stress) OR (posttraumatic stress)) OR (post traumatic stress)) OR (ptsd)**

**AND**

**2. Sample [All Field]**

**(general population) OR (representative) OR (population sample) OR (community sample)**

**AND**

**3. OECD High Income Country [Title/Abstract]**

**(australia) OR (australian)**

**OR**

**(austria) OR (austrian)**

**OR**

**(belgium) OR (belgian)**

**OR**

**(canada) OR (canadian)**

**OR**

**(chile) OR (chilean)**

**OR**

**((czech republic) OR (czechia)) OR (czech)**

**OR**

**(denmark) OR (danish)**

**OR**

**((estonia) OR (estonian)) OR (estland)**

**OR**

**(finland) OR (finnish)**

**OR**

**(france) OR (french)**

**OR**

**(germany) OR (german)**

**OR**

**(greece) OR (greek)**

**OR**

**(iceland) OR (icelandic)**

**OR**

**(ireland) OR (irish)**

**OR**

**(israel) OR (israeli)**

**OR**

**(italy) OR (italian)**

**OR**

**(japan) OR (japanese)**

**OR**

**((luxembourg) OR (luxembourgian)) OR (luxembourgish)**

**OR**

**((netherlands) OR (dutch)) OR (holland)**

**OR**

**new zealand**

**OR**

**(norway) OR (norwegian)**

**OR**

**(poland) OR (polish)**

**OR**

**(portugal) OR (portuguese)**

**OR**

**((slovakia) OR (slovak)) OR (slovakian)**

**OR**

**((slovenia) OR (slovene)) OR (slovenian)**

**OR**

**(south korea) OR (korean)**

**OR**

**((spain) OR (spanish)) OR (hispanic)**

**OR**

**(sweden) OR (swedish)**

**OR**

**(switzerland) OR (swiss)**

**OR**

**((((united kingdom) OR (uk)) OR (england)) OR (britain)**

**OR**

**((united states) OR (usa) OR (north-american)**

**AND NOT**

**4. Exclusion [Title/Abstract]**

**(((children) OR (child)) OR (adolescent)) OR (youth) OR (elderly) OR (old adults)**

**FILTER**

AND (clinicalstudy[Filter] OR clinicaltrial[Filter] OR comparativestudy[Filter] OR controlledclinicaltrial[Filter] OR journalarticle[Filter] OR meta-analysis[Filter] OR multicenterstudy[Filter] OR observationalstudy[Filter] OR pragmaticclinicaltrial[Filter] OR randomizedcontrolledtrial[Filter] OR review[Filter] OR systematicreview[Filter])

AND Year 1994-2022

**Search strings for post-traumatic stress disorder (PTSD) in the general population of OECD High-income countries exposed to either Natural disasters or Human-made Disasters**

**PubMed/ Medline**

**Keyword Natural Disaster [Title/Abstract]**

((((((((((((((((((((((((avalanche[Title/Abstract]) OR (landslide[Title/Abstract])) OR (earthquake[Title/Abstract])) OR (sinkhole[Title/Abstract])) OR (volcanic eruption[Title/Abstract])) OR (volcanoe[Title/Abstract])) OR (volcanic activity[Title/Abstract])) OR (flood[Title/Abstract])) OR (tsunami[Title/Abstract])) OR (cyclone[Title/Abstract])) OR (typhoon[Title/Abstract])) OR (hurricane[Title/Abstract])) OR (storm[Title/Abstract])) OR (tornado[Title/Abstract])) OR (blizzard[Title/Abstract])) OR (wildfire[Title/Abstract])) OR (bushfire[Title/Abstract])) OR (cold wave[Title/Abstract])) OR (heat wave[Title/Abstract])) OR (extreme temperature[Title/Abstract])) OR (natural disaster[Title/Abstract])) OR (natural catastrophe[Title/Abstract])) OR (flooding[Title/Abstract])) OR (extreme precipitation[Title/Abstract])) OR (heavy precipitation[Title/Abstract])

**OR**

**Keywords Human-made Disaster [Title/Abstract]**

(((((((((((((((((((((((((((((((((shooter[Title/Abstract]) OR (shooting[Title/Abstract])) OR (mass shooting[Title/Abstract])) OR (gunman[Title/Abstract])) OR (explosion[Title/Abstract])) OR (bomb[Title/Abstract])) OR (bombing[Title/Abstract])) OR (IED[Title/Abstract])) OR (improvised explosive devices[Title/Abstract])) OR (suicide vest[Title/Abstract])) OR (suicide attack[Title/Abstract])) OR (rocket attack[Title/Abstract])) OR (mortar attack[Title/Abstract])) OR (vehicle-based attack[Title/Abstract])) OR (vehicle-ramming attack[Title/Abstract])) OR (car attack[Title/Abstract])) OR (biological attack[Title/Abstract])) OR (chemical attack[Title/Abstract])) OR (radiological attack[Title/Abstract])) OR (stabbing[Title/Abstract])) OR (aircraft attack[Title/Abstract])) OR (aircraft hijacking[Title/Abstract])) OR (hijacking[Title/Abstract])) OR (kidnapping[Title/Abstract])) OR (vehicular assault[Title/Abstract])) OR (raid[Title/Abstract])) OR (hostage[Title/Abstract])) OR (hostage crisis[Title/Abstract])) OR (ambush[Title/Abstract])) OR (terrorism[Title/Abstract])) OR (terrorist incidents[Title/Abstract])) OR (attack[Title/Abstract])) OR (terrorist attack[Title/Abstract])) OR (massacre[Title/Abstract])

**AND**

**OECD High-Income Country [Title/Abstract]**

(((((((((((((((((((((((((((((((((((((((((((((((((((((((((((((((((((((((((australia[Title/Abstract]) OR (austria[Title/Abstract])) OR (belgium[Title/Abstract])) OR (canada[Title/Abstract])) OR (chile[Title/Abstract])) OR (czech republic[Title/Abstract])) OR (czechia[Title/Abstract])) OR (denmark[Title/Abstract])) OR (estonia[Title/Abstract])) OR (estland[Title/Abstract])) OR (finland[Title/Abstract])) OR (france[Title/Abstract])) OR (germany[Title/Abstract])) OR (greece[Title/Abstract])) OR (iceland[Title/Abstract])) OR (ireland[Title/Abstract])) OR (israel[Title/Abstract])) OR (italy[Title/Abstract])) OR (japan[Title/Abstract])) OR (luxembourg[Title/Abstract])) OR (new zealand[Title/Abstract])) OR (norway[Title/Abstract])) OR (poland[Title/Abstract])) OR (portugal[Title/Abstract])) OR (slovakia[Title/Abstract])) OR (slovenia[Title/Abstract])) OR (south korea[Title/Abstract])) OR (spain[Title/Abstract])) OR (sweden[Title/Abstract])) OR (switzerland[Title/Abstract])) OR (united kingdom[Title/Abstract])) OR (uk[Title/Abstract])) OR (england[Title/Abstract])) OR (britain[Title/Abstract])) OR (united states[Title/Abstract])) OR (usa[Title/Abstract])) OR (north-america[Title/Abstract])) OR (australia[MeSH Terms])) OR (austria[MeSH Terms])) OR (belgium[MeSH Terms])) OR (canada[MeSH Terms])) OR (chile[MeSH Terms])) OR (czech republic[MeSH Terms])) OR (czechia[MeSH Terms])) OR (denmark[MeSH Terms])) OR (estonia[MeSH Terms])) OR (estland[MeSH Terms])) OR (finland[MeSH Terms])) OR (france[MeSH Terms])) OR (germany[MeSH Terms])) OR (greece[MeSH Terms])) OR (iceland[MeSH Terms])) OR (ireland[MeSH Terms])) OR (israel[MeSH Terms])) OR (italy[MeSH Terms])) OR (japan[MeSH Terms])) OR (luxembourg[MeSH Terms])) OR (netherlands[MeSH Terms])) OR (new zealand[MeSH Terms])) OR (norway[MeSH Terms])) OR (poland[MeSH Terms])) OR (portugal[MeSH Terms])) OR (slovakia[MeSH Terms])) OR (slovenia[MeSH Terms])) OR (south korea[MeSH Terms])) OR (spain[MeSH Terms])) OR (sweden[MeSH Terms])) OR (switzerland[MeSH Terms])) OR (united kingdom[MeSH Terms])) OR (england[MeSH Terms])) OR (great britain[MeSH Terms])) OR (uk[MeSH Terms])) OR (united states[MeSH Terms])) OR (usa[MeSH Terms])

**AND**

**Syndrome [Title/Abstract]**

**(((post-traumatic stress) OR (posttraumatic stress)) OR (post traumatic stress)) OR (ptsd)**

**Exclusion [Title/Abstract]**

**(((children) OR (child)) OR (adolescent)) OR (youth) OR (elderly) OR (old adults)**

**AND FILTER**

Year 2000-2022
