# Supplementary material for: Work-related posttraumatic stress disorder in paramedics in comparison to data from the general population of working age. A systematic review and meta-analysis
Source: Front Public Health. 2023 Mar 9;11:1151248. doi: 10.3389/fpubh.2023.1151248 (PMC10035789; doi:10.3389/fpubh.2023.1151248)
Supplement: Supplementary file 1 [file Data_Sheet_1.ZIP › S5 Study Characteristics_Paramedics.docx]

Supplementary Table 1. Characteristics of included studies on PTSD in paramedics [1-39].

| **First Author, Yr public.** | **Design/**  **Country** | **Yr data collection** | **Target group** | **Sampling frame** | **Sample size** | **Type of trauma** | **Male (%)** | **Age yr (Mean)** | **Measure-ment/ Class Scheme** | **PTSD scale** | **Prev PTSD (%)** | **Relative rank CA** |
| --- | --- | --- | --- | --- | --- | --- | --- | --- | --- | --- | --- | --- |
| Alaqeel, 2019 | CS/ Saudi Arabia | 2018 | Paramedics, EMT | Employees of one level I trauma center | 49 | VE | 100 | NR | Screening/ DSM-IV | PCL-C | 26.5% | 0.765 |
| Alexander 2001^a^ | CS/ UK | 1999 | Paramedics, EMT | Employees of one local ambulance service | 90 | DE | 84 | NR | Screening/none | IES | 30.0% | 0.706 |
| Bennett, 2004^a^ | CS/ UK | 2002 | Paramedics, EMT | Employees of one local ambulance service | 293 | DE | 83.3 | 39.6 | Screening/DSM-IV | PDS | 22% | 0.647 |
| Brauchle, 2006 | LS/ Austria | 2000 | EMS personnel | Rescue workers involved in catastrophe of Kaprun 2000 | 35 | DE | 68.6 | 38.7 | Screening/DSM-IV | PDS | 5.7% | 0.471 |
| Carleton, 2018 | CS/ Canada | 2016 | Paramedics | Nationwide Canadian public safety personnel | 776 | DE | 67.4 | NR | Screening/DSM-5 | PCL-5 | 24.5% | 0.824 |
| Di Fiorino, 2004 | CS/ Italy | 2003 | Ambulance personnel | Ambulance personnel involved in Cardoso flood of 1996 | 34 | DE | 58.8 | 32.7 | Diagnosis/ DSM-IV | CAPS | 11.8% | 0.529 |
| Donnelly, 2020 | CS/ Canada | 2019 | Employees certified at least Primary Care Paramedic level | All EMS in the province Ontario | 717 | VE | 66.0 | 38.0 | Screening/ DSM-IV | PCL-M | 14.5% | 1 |
| Eiche, 2019 | CS/ Germany | 2017 | Paramedics | Paramedics any level from Germany nationwide EMS | 2684 | VE | 80.0 | (34.7) | Screening/ DSM-IV | Short DSM-IV Screening Scale PTSD | 5.7% | 0.882 |
| Feldman, 2021 | LS/ USA | 2019 | Paramedics, EMT | Employees of one local EMS | 135 | VE | 77.8 | 35.6 | Screening/ DSM-IV | PCL-C | 14.8% | 0.941 |
| Gallagher, 2009 | CS/ Ireland | 2008 | Mixed: EMT (85%) and dispatcher | Employees of one local EMS | 27 | VE | 100 | 40.0 | Screening/ DSM-IV | PCL-S | 44.4% | 0.647 |
| Häller, 2009 | CS/ German-speaking Switzerland and Liechtenstein | 2007 | Paramedics | Employees of all EMS in study region | 668 | DE | 66.9 | 36.3 | Screening/ DSM-IV | PDS | 4.3% | 0.882 |
| Halpern, 2010^a^ | CS/ Canada | 2008 | Ambulance personnel (front-line, supervisors) | Ambulance workers with mandatory education programme in local EMS | 228 | DE | 64.0 | 37.5 | Screening/none | IES-R | 8% | 0.647 |
| Hsiao, 2019 | LS/ Taiwan | 2016 | Paramedics (advanced EMT) | Paramedics involved in earthquake in 2016 | 38 | DE | 100 | 35.0 (Median) | Screening/ DSM-IV | PCL-C | 34.2% | 0.882 |
| Jasielska, 2019 | CS/ Poland | 2017 | Paramedics | Paramedics in State Medical Rescue System of province Wielkopolskie | 145 | VE | 66.2 | 31.0 | Screening/ none | IES-R | 48.9% | 0.471 |
| Jones, 2018 | CS/ USA | 2017 | Mixed: EMT, Paramedics (80%) and Firefighters | Firefighters and EMT/ paramedics from a federal state (Arkansas) | 195 | VE | 85.6 | NR | Screening/ DSM-IV | PCL-C | 25.6% | 0.824 |
| Jonsson, 2004^a^ | CS/ Sweden | 2002 | EMT | All ambulance emergency personnel of the region Vaestra Goetaland | 240 | DE | 79.2 | 38.0 | Screening/ none | IES | 12.1% | 0.706 |
| Katsavouni, 2016 | CS/ Greece | 2015 | Ambulance personnel (at least 1 year of work experience) | Ambulance personnel across Greece | 253 | DE | 73.9 | 36.4 | Screening/ DSM-IV | PTSS-10 | 13.8% | 0.529 |
| Khan, 2020a | CS/ Saudi-Arabia | 2018 | Paramedics | Paramedics from the Western region of Saudi Arabia | 104 | VE | 100 | 32.5 | Screening/ none | PSQI-A | 42% | 0.882 |
| Khan, 2020b | CS/ Australia | 2017 | Paramedics | Paramedics from a federal state (Victoria) | 83 | VE | 100 | 44.1 | Screening/ none | PSQI-A | 29% | 0.824 |
| Köhler, 2018 | CS/ Germany | 2017 | Mixed: Paramedics (83,2%) and firefighters | Emergency service personnel from 2 local EMS companies | 131 | DE | 77.1 | 36.6 | Screening/ none | IES-R | 16.8% | 0.882 |
| Kucmin, 2018 | CS/ Poland | 2016 | Paramedics | Paramedics in State Medical Rescue System in five provinces | 159 | VE | 87.4 | 34.1 | Screening/ none | IES-R | 27.7% | 0.647 |
| Kyron, 2021 | CS/ Australia | 2017 | Employees in emergency services | All ambulance, fire, and rescue, police, and state EMS organizations (National survey) | 3473 | VE | 53.2 | NR | Screening/ DSM-5 | PCL-5 | 8.2% | 0.882 |
| Luftman, 2017 | CS/ USA | 2015 | Paramedics, EMT | Four Trauma Regional Advisory Council ambulance systems in federal state Texas | 280 | VE | NR | NR | Screening/ none | PC-PTSD | 42.9% | 0.647 |
| Mishra, 2010^a^ | CS/ USA | 2007 | EMS personnel | EMS personnel working for the City and County of Honolulu | 101 | DE | 57.4 | NR | Screening/ DSM-IV-R | PCL-C | 4% | 0.706 |
| Ogińska-Bulik, 2021 | CS/ Poland | 2019 | Paramedics | 12 units of various state medical rescue stations, emergency medical teams, emergency wards, cancer wards, intensive care units, and hospices | 201 | VE | 60.2 | 39.6 | Screening/ DSM-5 | STSI | 43.3% | 0.882 |
| Oravecz, 2018 | CS/ Slovenia | 2013 | Paramedics | All EMS units registered with the Slovenian chamber of medical care | 347 | VE | 66.9 | NR | Screening/ DSM-IV-TR | PCL-M | 21% | 0.471 |
| Parobkiewicz, 2021 | CS/ Poland | 2019 | Paramedics | Paramedics in State Medical Rescue System of province Wielkopolskie | 106 | VE | 59.4 | 29.5 | Screening/ none | IES-R | 45% | 0.529 |
| Perrin, 2007^a^ | LS/ USA | 2003 | EMS, medical disaster personnel (WTC-HR) | All NYC and non-NYC EMS and medical organizations, disaster medical assistance teams in the aftermath of 9/11 2001 | 1741 | DE | 77.5^b^ | NR | Screening/ DSM-IV | PCL-C | 14.1% | 1 |
| Regehr, 2002^a^ | CS/ Canada | 2001 | Paramedics | Employees of a local EMS  organization | 86 | VE | NR | 39.7 | Screening/ none | IES | 25.5% | 0.706 |
| Reti, 2021 | CS/ New Zeeland | 2020 | Mixed: Paramedics 61%), patient transfer officers, dispatcher | Ambulance staff employed by one local EMS in Wellington | 125 | VE | 38.4 | 38.0 | Screening/ DSM-V | PCL-5 | 20% | 0.824 |
| Rybojad, 2016 | CS/ Poland | 2014 | Paramedics | Paramedics employed in prehospital medical emergency teams, transportation, hospital ED | 100 | DE | 86.0 | 33.6 | Screening/ none | IES-R | 40% | 0.647 |
| Setlack, 2021 | CS/ Canada | 2019 | Paramedics | Employees of one emergency service organization providing EMS and fire services within Winnipeg, Manitoba | 119 | DE | 75.6 | NR | Screening/ DSM-5 | PCL-5 | 18.5% | 0.765 |
| Shepherd, 2014 | CS/ UK | 2011 | Paramedics, EMT | Employees of one EMS: London Ambulance Service | 45 | VE | 68.9 | 37.0 | Screening/ DSM-IV | PDS | 15.5% | 0.765 |
| Straud, 2018 | CS/ USA | 2016 | Firefighter paramedics | Employees of one South Florida department  with four fire stations | 125 | VE | 86.4 | NR | Screening/ DSM-IV | PCL-C | 6.6% | 0.765 |
| Surgenor, 2020 | CS/ New Zealand | 2017 | Mixed: Paramedics, EMT (80,9%), clinical hub nurses, dispatcher | Employees of one nationwide paramedic agency providing front-line responses | 471 | VE | 51.8 | 42.3 | Screening/ DSM-5 | PCL-5 | 14.6% | 0.824 |
| Tatebe, 2020 | CS/ USA | 2019 | Paramedics | Paramedics of an urban Level I trauma unit | 94 | VE | 78.8^c^ | (40.8) | Screening/ DSM-5 | PCL-5 | 25% | 0.765 |
| Teegen, 2000 | CS/ Germany | 1998 | Paramedics, EMT | 10 EMS in Northern Germany | 129 | VE | 96.1 | 34.0 | Screening/ DSM-IV | PCL-C | 36% | 0.647 |
| Tremblay, 2020 | CS/ Canada | 2018 | Paramedics | Paramedics from a local EMS: Ambulance New Brunswick | 25 | VE | 68.0 | 38.5 | Screening/ DSM-IV | PCL-C | 16% | 0.765 |
| van der Ploeg, 2003^a^ | LS/ Netherlands | 2022 | Paramedics, drivers | All ambulance services in the Netherlands | 112 | DE | 85.7 | 39.8 | Screening/ none | IES | 13% | 0.824 |
| Yip, 2016a^a^ | LS/ USA | 2013 | EMS personnel | EMS personnel registered in NYC ambulance system and actively involved during 09/11 2001 | 1863 | DE | 81.0 | 36.2 (Median) | Screening/ DSM-IV | PCL-C | 7.9% | 0.882 |
| Yip, 2016b^a^ | LS/ USA | 2013 | EMS personnel | EMS personnel registered in NYC ambulance system and not involved during 09/11 2001 | 418 | VE | 67.9 | 36.2 (Median) | Screening/ DSM-IV | PCL-C | 2.4% | 0.882 |

*Note.* ^a^study used for Google Scholar citation tracking search. ^b^sample mean proportion based on raw data from all 29,572 rescue/recovery worker in the WTC-HR Wave 1 Adult Survey from https://www1.nyc.gov/site/911health/researchers/health-data-tools.page, ^c^sample mean proportion based on all 258 participants.

*Abbreviations.* CA: Critical appraisal. CAPS: Clinician administered PTSD scale. Class: Classification. CS: Cross-sectional study. DE: Discrete events. DSM: Diagnostic and Statistical Manual of Mental Disorders. ED: Emergency department. EMS: Emergency medical service. EMT: Emergency medical technician. ICD: International Classification of Diseases. IES (R): Impact of event scale (Revised). LS: Longitudinal study. NR: not reported. NYC: New York City. PCL-C: PTSD Checklist – Civilian Version. PCL-M: PTSD Check-List - Military Version. PCLS: Posttraumatic Stress Disorder Checklist scale. PCL-5: PTSD Checklist for DSM-5. PC-PTSD: Primary Care PTSD screen. PDS: Posttraumatic Diagnostic Scale. Prev: Prevalence. PSQI-A: Pittsburgh Sleep Quality Index-Addendum. PTSS-10: Post-Traumatic Stress Syndrome 10-Questions Inventory. STSI: Secondary Traumatic Stress Inventory. VE: Vague events, WTC-HR: World Trade Center Health Registry. Yr: year(s).

1. Alaqeel MK, Aljerian NA, AlNahdi MA, Almaini RY (2019) Post-traumatic stress disorder among Emergency Medical Services personnel: a cross-sectional study. Asian J Med Sci 10 (4):28-31. doi:10.3126/ajms.v10i4.23990

2. Alexander DA, Klein S (2001) Ambulance personnel and critical incidents: impact of accident and emergency work on mental health and emotional well-being. Br J Psychiatry 178 (1):76-81. doi:10.1192/bjp.178.1.76

3. Bennett P, Williams Y, Page N, Hood K, Woollard M, Vetter N (2005) Associations between organizational and incident factors and emotional distress in emergency ambulance personnel. Br J Clin Psychol 44 (Pt 2):215-226. doi:10.1348/014466505x29639

4. Brauchle G (2006) [Incidence and reaction-related predictors of the acute and posttraumatic stress disorder in disaster workers]. Z Psychosom Med Psychother 52 (1):52-62

5. Carleton RN, Afifi TO, Turner S, Taillieu T, Duranceau S, LeBouthillier DM, Sareen J, Ricciardelli R, MacPhee RS, Groll D, Hozempa K, Brunet A, Weekes JR, Griffiths CT, Abrams KJ, Jones NA, Beshai S, Cramm HA, Dobson KS, Hatcher S, Keane TM, Stewart SH, Asmundson GJG (2018) Mental disorder symptoms among public safety personnel in Canada. Can J Psychiatry 63 (1):54-64. doi:10.1177/0706743717723825

6. Di Fiorino M, Massimetti G, Nencioni M, Paoli RA (2004) [Post-Traumatic Stress Disorder seven years after a flooding in rescue squads]. Psichiatria e Territorio 21 (1):32-40

7. Donnelly EA, Bradford P, Davis M, Hedges C, Socha D, Morassutti P, Pichika SC (2020) What influences safety in paramedicine? Understanding the impact of stress and fatigue on safety outcomes. J Am Coll Emerg Physicians Open 1 (4):460-473. doi:10.1002/emp2.12123

8. Eiche C, Birkholz T, Jobst E, Gall C, Prottengeier J (2019) Well-being and PTSD in German emergency medical services – A nationwide cross-sectional survey. PLoS ONE 14 (7):1-13. doi:10.1371/journal.pone.0220154

9. Feldman TR, Carlson CL, Rice LK, Kruse MI, Beevers CG, Telch MJ, Josephs RA (2021) Factors predicting the development of psychopathology among first responders: A prospective, longitudinal study. Psychol Trauma 13 (1):75-83. doi:10.1037/tra0000957

10. Gallagher S, McGilloway S (2009) Experience of critical incident stress among ambulance service staff and relationship to psychological symptoms. Int J Emerg Ment Health 11 (4):235-248

11. Häller P, Michael T, Koechlin KB (2009) [PTSD among paramedics]. Verhaltensther Verhaltensmed 30 (4):403-417

12. Halpern J, Maunder RG, Schwartz B, Gurevich M (2011) Identifying risk of emotional sequelae after critical incidents. Emerg Med J 28 (1):51-56. doi:10.1136/emj.2009.082982

13. Hsiao YY, Chang WH, Ma IC, Wu CL, Chen PS, Yang YK, Lin CH (2019) Long-term PTSD risks in Emergency Medical Technicians who responded to the 2016 Taiwan Earthquake: a six-month observational follow-up study. Int J Environ Res Public Health 16 (24). doi:10.3390/ijerph16244983

14. Jasielska A, Ziarko M (2019) [General and specific individual post-traumatic stress disorder-related mechanisms among paramedics]. Med Pr 70 (1):53-66. doi:10.13075/mp.5893.00757

15. Jones S, Nagel C, McSweeney J, Curran G (2018) Prevalence and correlates of psychiatric symptoms among first responders in a Southern State. Arch Psychiatr Nurs 32 (6):828-835. doi:10.1016/j.apnu.2018.06.007

16. Jonsson A, Segesten K, Jonsson A, Segesten K (2004) Daily stress and concept of self in Swedish ambulance personnel. Prehosp Disaster Med 19 (3):226-234. doi:10.1017/s1049023x00001825

17. Katsavouni F, Bebetsos E (2016) The relationship between burnout, PTSD symptoms and injuries among ambulance personnel. Sport Sci 9 (1):7-13

18. Khan WAA, Conduit R, Kennedy GA, Abdullah Alslamah A, Ahmad Alsuwayeh M, Jackson ML (2020) Sleep and mental health among paramedics from Australia and Saudi Arabia: a comparison study. Clocks Sleep 2 (2):246-257. doi:10.3390/clockssleep2020019

19. Köhler M, Schäfer H, Goebel S, Pedersen A (2018) The role of disclosure attitudes in the relationship between posttraumatic stress disorder symptom severity and perceived social support among emergency service workers. Psychiatry Res 270:602-610. doi:10.1016/j.psychres.2018.10.049

20. Kucmin T, Kucmin A, Turska D, Turski A, Nogalski A (2018) Coping styles and dispositional optimism as predictors of post-traumatic stress disorder (PTSD) symptoms intensity in paramedics. Psychiatr Pol 52 (3):557-571. doi:10.12740/pp/68514

21. Kyron MJ, Rikkers W, Bartlett J, Renehan E, Hafekost K, Baigent M, Cunneen R, Lawrence D (2021) Mental health and wellbeing of Australian police and emergency services employees. Arch Environ Occup Health:1-11. doi:10.1080/19338244.2021.1893631

22. Luftman K, Aydelotte J, Rix K, Ali S, Houck K, Coopwood TB, Teixeira P, Eastman A, Eastridge B, Brown CV, Davis M (2017) PTSD in those who care for the injured. Injury 48 (2):293-296. doi:10.1016/j.injury.2016.11.001

23. Mishra S, Goebert D, Char E, Dukes P, Ahmed I (2010) Trauma exposure and symptoms of post-traumatic stress disorder in emergency medical services personnel in Hawaii. Emerg Med J 27 (9):708-711. doi:10.1136/emj.2009.080622

24. Ogińska-Bulik N, Gurowiec PJ, Michalska P, Kędra E (2021) Prevalence and predictors of secondary traumatic stress symptoms in health care professionals working with trauma victims: A cross-sectional study. PLoS ONE 16 (2):1-19. doi:10.1371/journal.pone.0247596

25. Oravecz R, Penko J, Suklan J, Krivec J (2018) Prevalence of post-traumatic stress disorder, symptomatology and coping strategies among Slovene medical emergency professionals. Sigurnost 60 (2):117-127

26. Parobkiewicz A, Ziarko M, Krawczyk J, Jasielska J (2021) Posttraumatic stress disorder symptoms in persons involved in road accidents and paramedics. J Med Sci 90 (2):91-96

27. Perrin M, DiGrande L, Wheeler K, Thorpe L, Farfel M, Brackbill R (2007) Differences in PTSD prevalence and associated risk factors among World Trade Center disaster rescue and recovery workers. Am J Psychiatry 164 (9):1385-1394

28. Regehr C, Goldberg G, Hughes J (2002) Exposure to human tragedy, empathy, and trauma in ambulance paramedics. Am J Orthopsychiatry 72 (4):505-513. doi:10.1037/0002-9432.72.4.505

29. Reti T, de Terte I, Stephens C (2021) Traumatic exposure, work-related stressors and gender as risk factors in the development of psychological distress for ambulance personnel. Traumatology. doi:10.1037/trm0000315

30. Rybojad B, Aftyka A, Baran M, Rzońca P (2016) Risk factors for posttraumatic stress disorder in Polish paramedics: a pilot study. J Emerg Med 50 (2):270-276. doi:10.1016/j.jemermed.2015.06.030

31. Setlack J, Brais N, Keough M, Johnson EA (2021) Workplace violence and psychopathology in paramedics and firefighters: mediated by posttraumatic cognitions. Can J Behav Sci 53 (3):211-220. doi:10.1037/cbs0000240

32. Shepherd L, Wild J (2014) Cognitive appraisals, objectivity and coping in ambulance workers: a pilot study. Emerg Med J 31 (1):41-44. doi:10.1136/emermed-2011-200511

33. Straud C, Henderson SN, Vega L, Black R, Van Hasselt V (2018) Resiliency and posttraumatic stress symptoms in firefighter paramedics: The mediating role of depression, anxiety, and sleep. Traumatology 24 (2):140-147. doi:10.1037/trm0000142

34. Surgenor LJ, Snell DL, Dorahy M (2020) Factors associated with psychiatric morbidity, post-traumatic stress disorder and post-traumatic growth in paramedic first responders: the role of core beliefs. Australasian J Paramed 17:1-8. doi:10.33151/ajp.17.727

35. Tatebe LC, Siva NR, Pekarek S, Liesen E, Wheeler A, Reese C, Schlanser V, Kaminsky M, Messer T, Starr F, Mis J, Bokhari F, Dennis A (2020) Heroes in crisis: Trauma centers should be screening for and intervening on post-traumatic stress in our emergency responders. J Trauma Acute Care Surg 89 (1):132-139. doi:10.1097/TA.0000000000002671

36. Teegen F, Yasui Y (2000) [Traumaexposure and posttraumatic stress disorder in emergency personnel]. Verhaltensther Verhaltensmed 21 (1):65-83

37. Tremblay M, Albert WJ, Fischer SL, Beairsto E, Johnson MJ (2020) Exploration of the health status of experienced New Brunswick paramedics. Work 66 (2):461-473. doi:10.3233/wor-203185

38. van der Ploeg E, Kleber RJ (2003) Acute and chronic job stressors among ambulance personnel: predictors of health symptoms. Occup Environ Med 60 (Suppl 1):i40-46. doi:10.1136/oem.60.suppl_1.i40

39. Yip J, Zeig-Owens R, Webber MP, Kablanian A, Hall CB, Vossbrinck M, Xiaoxue L, Weakley J, Schwartz T, Kelly KJ, Prezant DJ, Liu X (2016) World Trade Center-related physical and mental health burden among New York City Fire Department emergency medical service workers. Occup Environ Med 73 (1):13-20. doi:10.1136/oemed-2014-102601
