# Supplementary material for: Work-related posttraumatic stress disorder in paramedics in comparison to data from the general population of working age. A systematic review and meta-analysis
Source: Front Public Health. 2023 Mar 9;11:1151248. doi: 10.3389/fpubh.2023.1151248 (PMC10035789; doi:10.3389/fpubh.2023.1151248)
Supplement: Supplementary file 1 [file Data_Sheet_1.ZIP › S6 Study Characteristics_General_Pop.docx]

Supplementary Table 2. Characteristics of included studies on PTSD in the comparison groups [1-112].

| **First Author, Yr. public.** | **Design/ Country** | **Yr data collection** | **Target group**  **(definition)** | **Procedure** | **Sample size** | **Measurement/**  **Classification Scheme** | **PTSD measure** | **Prevalence PTSD** |
| --- | --- | --- | --- | --- | --- | --- | --- | --- |
| Andrews, 2001a | CS/ Australia | 1997 | GP UX | Australian National Survey of Mental Health and Well-Being (randomly drawn from private households across Australia) | 10,641 | Diagnosis/ ICD-10 | CIDI | 3.30% (351) |
| Andrews, 2001b | CS/ Australia | 1997 | GP UX |  | 10,641 | Diagnosis/ DSM-IV | CIDI | 1.30% |
| Beesdo-Baum, 2015 | LS/ Germany | 2003 | GP UX | Early Developmental Stages of Psychopathology Follow-up 3 (randomly drawn from population registries) | 2,210 | Diagnosis/ DSM-IV | DIA-X/M-CIDI | 1.36% |
| Ben-Ezra, 2018 | CS/ Israel | 2017 | GP UX | Stratified and randomly drawn sample from representative panel data of inhabitants administered by the Israeli Bureau of Statistics | 1,003 | Screening/ ICD-11 | ITQ | 8.97% |
| Bronner, 2009 | CS/ Netherlands | 2006 | GP UX | Stratified and randomly drawn sample from large access panel | 2,238 | Screening/ DSM-IV | SRS-PTSD | 3.75% |
| Bunting, 2013 | CS/ UK | 2004 | GP UX | Representative household survey as part of the WMH survey initiative | 1,986 | Diagnosis/ DSM-IV | WMH-CIDI | 6.50% |
| Cloitre, 2019 | CS/ USA | 2017 | GP UX | Randomly drawn from representative online research panel | 1,893 | Screening/ ICD-11 | ITQ | 3.28% (62) |
| de Girolamo, 2006 | CS/ Italy | 2001 | GP UX | Representative household survey as part of the WMH survey initiative | 4,712 | Diagnosis/ DSM-IV | WMH-CIDI | 0.80% |
| De Vries, 2009 | CS/ Netherlands | 2004 | GP UX | Nationally representative survey of inhabitants via random digit dialling | 1,087 | Diagnosis/ DSM-IV | CIDI | 3.3% |
| Erickson, 2013 | CS/ USA | 2010 | GP UX | Randomly drawn from US States Postal Service’s computerized Delivery Sequence File of Montana (Montana Health Matters study) | 3,360 | Screening/ DSM-IV | PCL-C | 4.22% |
| Faravelli, 2004 | CS/ Italy | 2002 | GP UX | Italian community sample randomly drawn from General practicioners working in Sesto Fiorentino (Tuscany) | 732 | Diagnosis/ DSM-IV | FPI | 0.14% |
| Frissa, 2013 | CS/ UK | 2008 | GP UX | Randomly selected households from user postcode address file (South East London Community Health study) | 1,698 | Screening/ DSM-IV | PC-PTSD | 5.48% |
| Goldstein, 2016 | CS/ USA | 2012 | GP UX | Representative US household survey (National Epidemiologic Survey on Alcohol and Related Conditions-III) | 36,309 | Diagnosis/ DSM-5 | AUDADIS-5 | 4.7% |
| Hapke, 2006 | CS/ Germany | 1996 | GP UX | Proportional random sample of individuals aged 18– 64 years in the cover-area of Luebeck | 4,075 | Diagnosis/ DSM-IV | M-CIDI | 0.71% |
| Hauffa, 2012 | CS/ Germany | 2007 | GP UX | Stratified and randomly drawn representative German household sample | 2,510 | Screening/ DSM-IV | PTDS | 2.94% |
| Heir, 2019 | CS/ Norway | 2015 | GP UX | National probability sample of Norwegian adult population constructed by Central National Register of Norway | 1,779 | Screening/ DSM-5 | PCL-5 | 6.30% |
| Hepp, 2006 | LS/ Switzerland | 1999 | GP UX | Representative community sample aged 19 prospectively followed between 1978 and 1999 (Zurich Cohort study) | 367 | Diagnosis/ DSM-IV | SPIKE | 0% |
| Husky, 2015 | CS/ France | 2001 | GP UX | Representative household survey as part of the WMH survey initiative | 1,436 | Diagnosis/ DSM-IV | WMH-CIDI | 2.29% |
| Hyland, 2021 | CS/ Ireland | 2019 | GP UX | National representative household sample using stratified, random probability sampling | 1,020 | Screening/ ICD-11 | ITQ | 5% |
| Ishikawa, 2016 | CS/ Japan | 2002 | GP UX | Representative household survey as part of the WMH survey initiative (WMH Japan survey) | 1,682 | Diagnosis/ DSM-IV | WMH-CIDI | 0.71% |
| Ishikawa, 2018 | CS/ Japan | 2013 | GP UX | Representative household survey as part of the WMH survey initiative (WMH Japan 2^nd^ survey) | 2,450 | Diagnosis/ DSM-IV | WMH-CIDI 3.0 | 0.20% |
| Ito, 2019 | LS/ Japan | 2016 | GP UX | Panelist pool representative of the Japanese population (National Survey for Stress and Health) | 40,000 | Screening/ DSM-5 | PCL-5 | 16.40% |
| Jacobi, 2015 | CS/ Germany | 2009 | GP UX | Stratified and randomly drawn representative German household sample (German Health Interview and Examination Survey – Part 1) | 5,303 | Diagnosis/ DSM-IV | DIA-X/M-CIDI | 2.04% |
| Johns, 2012 | LS/ USA | 2008 | GP UX | Probability sample of households within a US metropolitan region (Detroit Neighborhood Health Study) | 1,221 | Screening/ DSM-IV | PCL-C | 10.89% |
| Karatzias, 2019 | CS/ UK | 2018 | GP UX | Randomly drawn from representative UK online research panel | 1,051 | Screening/ ICD-11 | ITQ | 5.33% |
| Karg, 2012 | CS/ USA | 2008 | GP UX | Subsample of a randomly drawn representative household sample (National Survey on Drug Use and Health) | 5,653 | Diagnosis/ DSM-IV | SCID-I | 0.71% |
| Kessler, 2005 | CS/ USA | 2001 | GP UX | Representative household survey as part of the WMH survey initiative | 5,692 | Diagnosis/ DSM-IV | WMH-CIDI | 3.51% |
| Kim, 2021 | CS/ South Korea | 2016 | GP UX | Stratified and randomly drawn representative household sample (2016 Korean Epidemiologic Catchment Area Study) | 5,075 | Diagnosis/ DSM-IV | CIDI | 1.75% |
| Knipscheer, 2020 | LS/ Netherlands | 2007 | GP UX | Stratified and randomly drawn representative Dutch household sample (Netherlands Mental Health Survey and Incidence Study-2) | 4,667 | Screening/ DSM-IV | TSQ | 1.99% |
| Koenen 2017a | CS/ Belgium | 2001 | GP UX | Representative household survey as part of the WMH survey initiative | 1,043 | Diagnosis/ DSM-IV | WMH-CIDI | 2.11% |
| Koenen 2017b | CS/ Germany | 2002 | GP UX | Representative household survey as part of the WMH survey initiative | 1,323 | Diagnosis/ DSM-IV | WMH-CIDI | 1.89% |
| Koenen 2017c | CS/ Portugal | 2008 | GP UX | Representative household survey as part of the WMH survey initiative | 2,060 | Diagnosis/ DSM-IV | WMH-CIDI | 3.74% |
| Koenen 2017d | CS/ Netherlands | 2002 | GP UX | Representative household survey as part of the WMH survey initiative | 1,093 | Diagnosis/ DSM-IV | WMH-CIDI | 4.57% |
| Lassemo, 2017 | CS/ Norway | 1989 | GP UX | CS-Data from the longitudinal representative OsLof study examining an urban (Oslo) and a rural (Lofoten) community | 1,634 | Diagnosis/ ICD-10 | CIDI 1.1 | 1.29% |
| Leray, 2011 | CS/ France | 1999 | GP UX | Representative national survey of the French adult population (Mental Health in General Population Survey) | 36,105 | Diagnosis/ ICD-10 | MINI | 0.70% |
| Levinson, 2007 | CS/ Israel | 2003 | GP UX | Representative household survey as part of the WMH survey initiative | 4,859 | Diagnosis/ DSM-IV | WMH-CIDI | 0.47% |
| Lukaschek, 2013 | LS/ Germany | 2006 | GP UX | Random selected from population registries (Cooperative Health Research in the Region of Augsburg F4 study) | 3,080 | Screening/ none | IES + hyper-arousal via interview | 1.66% |
| Maercker, 2008 | CS/ Germany | 2005 | GP UX | Stratified and random-route method to establish a representative household sample | 2,426 | Screening/ DSM-IV | PSS | 2.31% |
| Maercker, 2018 | CS/ Germany | 2016 | GP UX | Stratified and randomly drawn representative household sample | 2,524 | Screening/ ICD-11 | ITQ | 1.47% |
| McManus, 2007 | CS/ UK | 2006 | GP UX | Multi-stage stratified probability sampling using Postcode Address Files of households all over England (Adult Psychiatric Morbidity Survey) | 7,207 | Screening/ ICD-10 | TSQ | 3.01% |
| Miller, 2013 | CS/ USA | 2011 | GP UX | Probability-based online panel maintained by Survey Sampling International (National Stressful Events Survey) | 2,953 | Screening/ DSM-5 (original criteria) | PCL-5 | 5.38% |
| Muldoon, 2007 | CS/ Ireland | 2004 | GP UX | Random household samples of the population in Northern Ireland and the border counties of the Irish Republic | 3,000 | Screening/ DSM-IV | PCL | 9.97% |
| Navarro-Mateu, 2015 | CS/ Spain | 2010 | GP UX | Representative household survey as part of the WMH survey initiative (The PEGASUS-Murcia project) | 2,621 | Diagnosis/ DSM-IV | WMH-CIDI 3.0 | 0.99% |
| Ohayon, 2000 | CS/ Canada | 1996 | GP UX | Random representative household sample from a metropolitan area (Toronto) | 1,832 | Diagnosis/ DSM-IV | Diagnostic Interview | 1.86% |
| Olaya, 2015 | CS/ Spain | 2001 | GP UX | Representative household survey as part of the WMH survey initiative | 2,121 | Diagnosis/ DSM-IV | WMH-CIDI 3.0 | 0.61% |
| Parto, 2011 | LS/ USA | 2002 | GP UX | Probability sample of urban-residents (African-Americans and whites) (Healthy  Aging in Neighborhoods of Diversity across the Lifespan study) | 2,104 | Screening/ DSM-IV | PCL-C | 12.74% |
| Sareen, 2007 | LS/ Canada | 2002 | GP UX | Stratified, multistage clusters of households of 10 provinces representative GP (Canadian Community Health Survey cycle 1.2) | 36,984 | Diagnosis/ NR | Diagnosis of current PTSD by healthcare profession-al | 1.29% |
| Slade, 2009 | CS/ Australia | 2007 | GP UX | Stratified, multistage probability samples of households (2007 National Survey of Mental Health and Wellbeing survey) | 8,841 | Diagnosis/ ICD-10 | WMH-CIDI | 6.40% |
| Spitzer, 2009 | LS/ Germany | 2002 | GP UX | Two-stage representative cluster sampling of households in West Pomerania (Study of Health in Pomerania) | 3,171 | Diagnosis/ DSM-IV | SCID | 1.96% |
| Van Ameringen, 2008 | CS/ Canada | 2002 | GP UX | Nationally representative sample of households obtained by random digit dialing | 2,991 | Diagnosis/ DSM-IV | WMH-CIDI | 2.41% |
| van der Velden, 2018 | LS/ Netherlands | 2012 | GP UX | Random sample drawn from the population register by Statistics Netherlands (Longitudinal Internet Studies for the Social Sciences-panel ) | 1,798 | Screening/ DSM-IV | IES with hyper-arousal items IES-R | 2.84% |
| Wells, 2006 | CS/ New Zealand | 2003 | GP UX | Representative household survey as part of the WMH survey initiative (Te Rau Hinengaro: The New Zealand Mental Health  Survey) | 7,435 | Diagnosis/ DSM-IV | WMH-CIDI 3.0 | 3.0% |
| White, 2015a | LS/ UK | 2008 | GP UX | Stratified random sample of households in a borough in Wales (Caerphilly Health and Social Needs study) | 1,971 | Screening/ DSM-5 | TSQ | 8.12% |
| White, 2015b | LS/ UK | 2008 | GP UX |  | 1,971 | Screening/ DSM-IV | TSQ | 14.31% |
| Yoo, 2018 | CS/ South Korea | 2011 | GP UX | Stratified and randomly drawn representative household sample (2011 Korean Epidemiologic Catchment Area Study) | 5,909 | Diagnosis/ DSM-IV-R | Korean version of CIDI | 1.29% |
| Abeldano, 2014 | CS/ Chile | 2010 | GP ND | People affected by the 2010 earthquake in Chile (taken from stratified randomly selected household sample known as National Socioeconomic Characterization Survey) | 24,982 | Screening/ DSM-IV | DTS | 11.13% |
| Acierno, 2007 | CS/ USA | 2005 | GP ND | 33 Florida counties in direct path of hurricanes in 2004. Sample selected via random-digit dialing | 1,452 | Screening/ DSM-IV | NWS-PTSD Module | 3.58% |
| Agyapong, 2019 | CS/ Canada | 2016 | GP ND | Randomly selected adult inhabitants living in Fort McMurray during the 2016 Fort McMurray wildfire | 486 | Screening/ DSM-5 | PCL-5 | 12.76% |
| Alderman, 2013 | CS/ Australia | 2011 | GP ND | Random sample of households from 12 electorates affected by Summer Floods in Brisbane | 99 | Screening/ DSM-5 | PCL-C | 16.16% |
| Belleville, 2021 | CS/ Canada | 2017 | GP ND | Randomly selected adult inhabitants evacuated from Fort McMurray during the 2016 Fort McMurray wildfire | 1,510 | Screening/ DSM-5 | PCL-5 | 15.43% |
| Boscarino, 2013 | CS/ USA | 2013 | GP ND | Randomly selected households from communities affected by 2012 Hurricane Sandy | 200 | Screening/ DSM-IV | PC-PTSD | 14.5% |
| Bryant, 2014 | LS/ Australia | 2011 | GP ND | Randomly selected adults living in communities affected by the Victorian Black Saturday bushfires | 812 | Screening/ DSM-IV | PC-PTSD | 13.55% |
| Carlsen, 2012 | CS/ Iceland | 2010 | GP ND | All inhabitants living in the area of ash fall due to volcanic eruption (Eyjafjallajökull, Iceland) | 138 | Screening/ DSM-IV | PSS | 7.25% |
| Catapano, 2001 | CS/ Italy | 1999 | GP ND | Stratified and randomly selected sample of adults affected by 1998 Landslide in Sarno | 272 | Screening/ DSM-IV | SRS-PTSD | 27.57% |
| Cerda, 2013 | LS/ USA | 2008 | GP ND | Stratified and randomly selected sample of households in counties affected by 2008 Hurricane Ike | 658 | Screening/ DSM-IV-TR | PCL-C | 6.08% |
| Dell'Osso, 2012 | CS/ Italy | 2009 | GP ND | All inhabitants living in the town L’Aquila during the 2009 L’Aquila earthquake | 900 | Screening/ DSM-IV-TR | TALS-SR | 41.33% |
| Di Fiorino, 2005 | CS/ Italy | 2003 | GP ND | All inhabitants living in Cardoso during the 1996 Versilia flooding | 61 | Screening/ DSM-IV | DTS | 45.90% |
| Fergusson, 2014 | LS/ New Zealand | 2012 | GP ND | Part of the panel of the Christchurch Health and Development Study affected by the 2010 Canterbury earthquakes | 543 | Diagnosis/ DSM-IV | CIDI | 4.42% |
| Flores, 2020 | CS/ USA | 2017 | GP ND | Randomly selected adults from the metropolitan statistical area of Houston at the time of 2017 Hurricane Harvey | 403 | Screening/ DSM-IV | PCL-S | 16.63% |
| French, 2019 | CS/ UK | 2016 | GP ND | Randomly selected sample of households affected by the 2015/16 floods (English National Study of Flooding and Health) | 412 | Screening/ DSM-IV | PCL-6 | 19.17% |
| Galea, 2007 | CS/ USA | 2006 | GP ND | 1^st^ Probability sample of households (via random-digit dialing) from areas affected by 2005 Hurricane Katrina; and sample of FEMA-supported evacuees | 1,043 | Screening/ DSM-IV | TSQ | 16.30% |
| Galea, 2008 | CS/ USA | 2007 | GP ND | Probability sample of adults living in the 23 southernmost counties of Mississippi prior to 2005 Hurricane Katrina (Hurricane Katrina Community Advisory Group) | 810 | Diagnosis/ DSM-IV | CIDI | 15.19% |
| Gigantesco, 2013 | CS/ Italy | 2010 | GP ND | Random sample of individuals from the Healthcare Register of two Local Health Units of L’Aquila and its province (2009 L’Aquila earthquake) | 957 | Diagnosis/ DSM-IV | MINI | 4.08% |
| Gissurardottir, 2019 | LS/ Iceland | 2010 | GP ND | All inhabitants living in the area close to the Eyjafjallajökull volcano at the time of eruption (prospective cohort study) | 1,146 | Screening/ DSM-IV | PC-PTSD | 6.20% |
| Heir, 2009 | LS/ Norway | 2004 | GP ND | All adult Norwegian nationals who were at a disaster stricken area of Thailand during the 2004 South-East Asia tsunami | 416 | Screening/ none | IES-R | 36.30% |
| Heo, 2008 | CS/ South Korea | 2008 | GP ND | Inhabitants participating in a national survey and living in a village during a massive flood in 2006 | 58 | Screening/ none | IES-R and MMPI-PTSD | 22.41% |
| Houston, 2015 | CS/ USA | 2012 | GP ND | Survey 1 data of a random sample of inhabitants of the city Joplin at the time of the 2011 Joplin, Missouri tornado | 380 | Screening/ DSM-IV | TSQ | 12.63% |
| Kato, 1998 | CS/ Japan | 1996 | GP ND | All evacuees living in shelters (100 temporary housing communities) after the 1995 Great Hanshin–Awaji Earthquake | 5,570 | Screening/ DSM-IV | PTSS-10 | 24.90% |
| Kukihara, 2014 | CS/ Japan | 2011 | GP ND | Inhabitants of a town located within 20 km radius of the Fukushima Nuclear Power Plant taken from a larger survey of the Japanese Ministry of Education (2011 Great East Japan Earthquake and Tsunami) | 241 | Screening/ none | IES-R | 33.20% |
| Lowe, 2015 | CS/ USA | 2013 | GP ND | Random sample of inhabitants of areas of NYC affected by 2012 Hurricane Sandy | 418 | Screening/ DSM-5 | PCL-5 | 1.91% |
| Marshall, 2007 | CS/ USA | 2003 | GP ND | All individuals evacuated and living in two facilities that provided EMS due to the 2003 California wildfires | 234 | Screening/ DSM-IV | PCL | 23.93% |
| Mason, 2010 | CS/ UK | 2008 | GP ND | All flood-affected households of two local councils in the UK | 444 | Screening/ DSM-IV | HTQ-R | 27.93% |
| Matthews, 2020 | CS/ Australia | 2017 | GP ND | Local community-partnered purposive snowball sampling technique to achieve inhabitants affected by heavy flooding in Northern New South Wales in 2017 | 1,888 | Screening/ DSM-IV | PCL-6 | 15.41% |
| McLaughlin, 2011 | LS/ USA | 2006 | GP ND | 2^nd^ and 3^rd^ subsample of a probability sample of inhabitants of affected areas by 2005 Hurricane Katrina (Hurricane Katrina Community Advisory Group) | 901 | Screening/ DSM-IV | TSQ | 17.09% |
| Norris, 2010 | CS/ USA | 2008 | GP ND | Disproportionate stratified cluster sampling of inhabitants living in the most damaged counties at the time of 2008 Hurricane Ike (Galveston Bay Recovery Study) | 658 | Screening/ DSM-IV | PCL-C | 7.45% |
| Paranjothy, 2011 | CS/ UK | 2007 | GP ND | Stratified and randomly selected sample of households of counties affected to the 2007 summer floods in England | 2,019 | Screening/ DSM-IV | PCL-C | 6.84% |
| Parslow, 2006 | LS/ Australia | 1999 | GP ND | Young adults from the PATH Through Life Project and experiencing a large bushfire in 2003 | 2,085 | Screening/ DSM-IV | TSQ | 4.99% |
| Priebe, 2009 | CS/ Italy | 2003 | GP ND | Randomly selected inhabitants from the population register of most affected villages by the 2002 Molise earthquake | 1,680 | Screening/ DSM-IV | Short screening scale for DSM-IV PTSD | 14.46% |
| Priebe, 2011 | CS/ Italy | 2005 | GP ND | Randomly selected inhabitants from the population register of a village affected to an earthquake in 1997 | 200 | Diagnostic/ DSM-IV | MINI | 0.5% |
| Ruggiero, 2012 | CS/ USA | 2009 | GP ND | Randomly recruited households from most affected counties by 2008 Hurricane Ike as part of a larger study on post-disaster intervention (National Institute of Mental Health study | 256 | Screening/ DSM-IV | NWS PTSD module | 3.91% |
| Shigemura, 2012 | CS/ Japan | 2011 | GP ND | All full-time nuclear power plant workers from the Daiichi and Daini plants during the 2011 Great East Japan Earthquake and Tsunami | 1,495 | Screening/ none | IES-R | 25.28% |
| Tsujiuchi, 2016 | CS/ Japan | 2012 | GP ND | All households of Fukushima evacuees living at Saitama prefecture during the 2011 Great East Japan Earthquake and Tsunami | 350 | Screening/ none | IES-R | 59.43% |
| Waite, 2017 | CS/ UK | 2015 | GP ND | Randomly selected sample of households affected by 2013/14 flooding in England (English National Study of Flooding and Health) | 1,925 | Screening/ DSM-IV | PCL-6 | 20.57% |
| Yabe, 2014 | LS/ Japan | 2012 | GP ND | Officially registered inhabitants from the nationally designated evacuation zones after Fukushima nuclear disaster in connection to the Great East Japan Earthquake and Tsunami (Fukushima Health Management survey 2011) | 60,704 | Screening/ DSM-IV | PCL-S | 21.60% |
| Adams, 2005 | CS/ USA | 2002 | GP HMD | Random sample of English- or Spanish–speaking adults living in NYC at the time of the attacks on the WTC on 9/11 2001 | 2,180 | Screening/ DSM-IV | NWS PTSD module | 5.09% |
| Besser, 2009 | CS/ Israel | 2007 | GP HMD | Stratified probability sample of adults living in Gaza-bordering communities, exposed to repeated missile attacks | 160 | Screening/ DSM-IV | IES-R | 26.88% |
| Chipman, 2011 | CS/ Israel | 2008 | GP HMD | Stratified and randomly selected sample of adults from the Israeli telephone database exposed to missile attacks | 1,001 | Screening/ DSM-IV | PSS | 5.49% |
| DeLisi, 2003 | CS/ USA | 2001 | GP HMD | Stratified representative sample of NYC inhabitants during the terrorist attacks on the WTC on 9/11 2001 | 1,009 | Screening/ DSM-IV | DTS | 18.53% |
| DiGrande, 2008 | CS/ USA | 2003 | GP HMD | Sample of adults with permanent residence south of Canal Street during the terrorist attacks on the WTC on 9/11 2001 taken from the WTC-HR | 11,037 | Screening/ DSM-IV | PCL-C | 12.60% |
| DiGrande, 2011 | CS/ USA | 2003 | GP HMD | Sample of WTC-tower survivors during the terrorist attacks on the WTC on 9/11 2001 taken from the WTC-HR | 3,271 | Screening/ DSM-IV | PCL-S | 15.04% |
| Elklit, 2007 | CS/ Denmark | 2005 | GP HMD | All evacuated inhabitants from Kolding after the 2004 firework factory explosion | 516 | Screening/ DSM-IV | HTQ-IV | 12.40% |
| Fagan, 2003 | CS/ USA | 2002 | GP HMD | Representative household sample via random digit dialing from the NYC metropolitan area affected by the terrorist attacks on the WTC on 9/11 2001 | 364 | Screening/ DSM-IV | NWS PTSD module | 8.24% |
| Farfel, 2008 | CS/ USA | 2003 | GP HMD | All individuals enrolled in the WTC-HR in the aftermath of the terrorist attacks on the WTC on 9/11 2001 (baseline data) | 68,444 | Screening/ DSM-IV | PCL-C | 15.62% |
| Ferrando, 2011 | CS/ Spain | 2004 | GP HMD | Stratified randomly selected sample of inhabitants of the Madrid suburb where the train bombs exploded in 2004 | 485 | Screening/ DSM-IV | DTS | 12.37% |
| Galea, 2002 | CS/ USA | 2001 | GP HMD | Representative sample of households south of 110th Street via random digit dialing affected by the terrorist attacks on the WTC on 9/11 2001 | 988 | Screening/ DSM-IV | NWS PTSD module | 7.49% |
| Genereux, 2019 | CS/ Canada | 2015 | GP HMD | Randomly selected sample of adults from the Eastern Townships Population Health Survey (2^nd^ wave) living in Lac-Mégantic and Granit area during the 2013 Lac-Mégantic Train Derailment Tragedy | 800 | Screening/ none | IES | 24.25% |
| Hafstad, 2017a^a^ | LS/ Norway | 2011 | GP HMD | All parents of adolescent survivors of the 2011 Norway attacks | 451 | Diagnosis/ DSM-5 | UCLA PTSD-RI for DSM-5 | 6.43% |
| Hafstad, 2017b^a^ | LS/ Norway | 2011 | GP HMD |  | 451 | Diagnosis/ ICD-11 | UCLA PTSD-RI for ICD-11 | 3.77% |
| Jose, 2018 | LS/ USA | 2013 | GP HMD | Inhabitants from the Boston metropolitan area part of a nationally representative longitudinal study on the 2013 Boston Marathon Bombings (Wave 2) | 657 | Screening/ DSM-IV | PCPS | 14.92% |
| Miguel-Tobal, 2006 | CS/ Spain | 2004 | GP HMD | Randomly selected household sample of Madrid city residents exposed to the 2004 train bombing explosions | 1,589 | Screening/ DSM-IV | NWS PTSD module | 2.33% |
| Nandi, 2005 | CS/ USA | 2002 | GP HMD | Representative sample of inhabitants via random digit dialing living in NYC 4 month after the terrorist attacks on the WTC on 9/11 2001 | 2,001 | Screening/ DSM-IV | NWS PTSD module | 7.40% |
| Riviere, 2008 | CS/ France | 2003 | GP HMD | Randomly drawn households from the national population census database of inhabitants of Toulouse exposed to the 2001 AZF chemical factory explosion | 811 | Screening/ none | IES-R | 14.06% |
| Schlenger, 2002 | CS/ USA | 2001 | GP HMD | Probability-based online research panel with households located in NYC during the terrorist attacks on the WTC on 9/11 2001(National Study of Americans’ Reactions to September 11) | 777 | Screening/ DSM-IV | PCL-C | 11.20% |
| Shalev, 2006 | CS/ Israel | 2001 | GP HMD | Randomly selected inhabitants from one suburb of Jerusalem, which is directly exposed to acts of terrorism | 167 | Screening/ DSM-IV | PSS | 26.95% |
| Stein, 2013 | CS/ Israel | 2009 | GP HMD | Stratified and randomly chosen phone numbers of residents of two communities exposed to frequent rocket attacks | 450 | Screening/ DSM-IV | PCL-C | 25.56% |
| Tracy, 2008 | LS/ Israel | 2004 | GP HMD | Randomly selected national sample of adults exposed to repeated acts of terrorism in Israel (baseline) | 1,613 | Screening/ DSM-IV | PSS | 8.93% |

| *Note:* ^a^Contrary to the definition of sampling frames used for study selection (i.e. area probability household samples, administrative list sampling) this study focused on indirect exposure to a terroristic attack. *Abbreviations.* AUDADIS-5: Alcohol Use Disorder and Associated Disabilities Interview Schedule—Diagnostic and Statistical Manual of Mental Disorders—Fifth Edition Version. CCHS: Canadian Community Health Survey. CIDI: Composite International Diagnostic Interview. CS: Cross-sectional study. DIA-X/M-CIDI: Diagnostisches Expertensystem für psychische Störungen/Munich-Composite International Diagnostic Interview. DSM: Diagnostic and Statistical Manual of Mental Disorder. DTS: Davidson Trauma Scale. EMS: Emergency medical services. FPI: Florence Psychiatric Interview. GP: General population. HMD: Human-made disaster. HTQ-IV: Harvard Trauma Questionnaire. ICD: International Classification of Diseases. IES (R): Impact of event scale (Revised). ITQ: International Trauma Questionnaire. LS: Longitudinal study. M-CIDI: German modified extented and DSM-IV adapted version of the WHO CIDI. NWS-PTSD Module: National Women’s Study PTSD Module. MINI: Mini International Neuropsychiatric Interview. MMPIPTSD: The PTSD domain of the Minnesota Multiphasic Personality Inventory. ND: Natural disaster. NYC: New York City. PCL: PTSD Checklist. PCL-C: PTSD Checklist – Civilian Version. PCL-S: PTSD Checklist – Specific. PCL-5: PTSD Checklist for DSM-5. PCL-6: Short-form of PTSD checklist. PCPS: Primary Care PTSD Screener. PC-PTSD: Primary Care PTSD screen. PSS: Posttraumatic symptom scale. PTDS: Posttraumatic Diagnostic Scale. PTSD: Post-traumatic stress disorder. PTSS-10: Post-Traumatic Stress Syndrome 10-Questions Inventory. SPIKE: Structured Psychopathological Interview and Rating of the Social Consequences for Epidemiology. SRS-PTSD: Self-Rating Scale for Post-Traumatic Stress Disorder. TALS-SR: Trauma and Loss Spectrum-Self Report. TSQ: Traumatic Screening Questionnaire. UCLA PTSD-RI: The University of California at Los Angeles PTSD Reaction Index. UX: unexposed. WMH: World Mental Health Survey Initiative WHO: World Health Organization. Composite International Diagnostic Interview. WTC: World Trade Center, WTC-HR: World Trade Center Health Register. |
| --- |

Reference List

1. Andrews G, Henderson S, Hall W (2001) Prevalence, comorbidity, disability and service utilisation. overview of the Australian National Mental Health Survey. Br J Psychiatry 178 (2):145-153

2. Beesdo-Baum K, Knappe S, Asselmann E, Zimmermann P, Bruckl T, Hofler M, Behrendt S, Lieb R, Wittchen HU (2015) The 'Early Developmental Stages of Psychopathology (EDSP) study': a 20-year review of methods and findings. Soc Psychiatry Psychiatr Epidemiol 50 (6):851-866. doi:10.1007/s00127-015-1062-x

3. Ben-Ezra M, Karatzias T, Hyland P, Brewin CR, Cloitre M, Bisson JI, Roberts NP, Lueger-Schuster B, Shevlin M (2018) Posttraumatic stress disorder (PTSD) and complex PTSD (CPTSD) as per ICD-11 proposals: A population study in Israel. Depress Anxiety 35 (3):264-274. doi:10.1002/da.22723

4. Bronner MB, Peek N, Vries M, Bronner AE, Last BF, Grootenhuis MA (2009) A community-based survey of posttraumatic stress disorder in the Netherlands. J Trauma Stress 22 (1):74-78. doi:10.1002/jts.20379

5. Bunting BP, Ferry FR, Murphy SD, O'Neill SM, Bolton D (2013) Trauma associated with civil conflict and posttraumatic stress disorder: evidence from the Northern Ireland study of health and stress. J Trauma Stress 26 (1):134-141. doi:10.1002/jts.21766

6. Cloitre M, Hyland P, Bisson JI, Brewin CR, Roberts NP, Karatzias T, Shevlin M (2019) ICD-11 Posttraumatic Stress Disorder and Complex Posttraumatic Stress Disorder in the United States: A Population-Based Study. J Trauma Stress 32 (6):833-842. doi:10.1002/jts.22454

7. de Girolamo G, Polidori G, Morosini P, Scarpino V, Reda V, Serra G, Mazzi F, Alonso J, Vilagut G, Visona G, Falsirollo F, Rossi A, Warner R (2006) Prevalence of common mental disorders in Italy: results from the European Study of the Epidemiology of Mental Disorders (ESEMeD). Soc Psychiatry Psychiatr Epidemiol 41 (11):853-861. doi:10.1007/s00127-006-0097-4

8. de Vries GJ, Olff M (2009) The lifetime prevalence of traumatic events and posttraumatic stress disorder in the Netherlands. J Trauma Stress 22 (4):259-267. doi:10.1002/jts.20429

9. Erickson LD, Hedges DW, Call VR, Bair B (2013) Prevalence of and factors associated with subclinical posttraumatic stress symptoms and PTSD in urban and rural areas of Montana: a cross-sectional study. J Rural Health 29 (4):403-412. doi:10.1111/jrh.12017

10. Faravelli C, Abrardi L, Bartolozzi D, Cecchi C, Cosci F, D'Adamo D, Lo Iacono B, Ravaldi C, Scarpato MA, Truglia E, Rossi Prodi PM, Rosi S (2004) The Sesto Fiorentino study: point and one-year prevalences of psychiatric disorders in an Italian community sample using clinical interviewers. Psychother Psychosom 73 (4):226-234. doi:10.1159/000077741

11. Frissa S, Hatch SL, Gazard B, Fear NT, Hotopf M (2013) Trauma and current symptoms of PTSD in a South East London community. Soc Psychiatry Psychiatr Epidemiol 48 (8):1199-1209. doi:10.1007/s00127-013-0689-8

12. Goldstein RB, Smith SM, Chou SP, Saha TD, Jung J, Zhang H, Pickering RP, Ruan WJ, Huang B, Grant BF (2016) The epidemiology of DSM-5 posttraumatic stress disorder in the United States: results from the National Epidemiologic Survey on Alcohol and Related Conditions-III. Soc Psychiatry Psychiatr Epidemiol 51 (8):1137-1148. doi:10.1007/s00127-016-1208-5

13. Hapke U, Schumann A, Rumpf HJ, John U, Meyer C (2006) Post-traumatic stress disorder: the role of trauma, pre-existing psychiatric disorders, and gender. Eur Arch Psychiatry Clin Neurosci 256 (5):299-306. doi:10.1007/s00406-006-0654-6

14. Hauffa R, Rief W, Brähler E, Martin A, Mewes R, Glaesmer H (2011) Lifetime traumatic experiences and posttraumatic stress disorder in the German population: results of a representative population survey. J Nerv Ment Dis 199 (12):934-939. doi:10.1097/NMD.0b013e3182392c0d

15. Heir T, Bonsaksen T, Grimholt T, Ekeberg Ø, Skogstad L, Lerdal A, Schou-Bredal I (2019) Serious life events and post-traumatic stress disorder in the Norwegian population. BJPsych Open 5 (5):e82. doi:10.1192/bjo.2019.62

16. Hepp U, Gamma A, Milos G, Eich D, Ajdacic-Gross V, Rössler W, Angst J, Schnyder U (2006) Prevalence of exposure to potentially traumatic events and PTSD. The Zurich Cohort Study. Eur Arch Psychiatry Clin Neurosci 256 (3):151-158. doi:10.1007/s00406-005-0621-7

17. Husky MM, Lépine JP, Gasquet I, Kovess-Masfety V (2015) Exposure to Traumatic Events and Posttraumatic Stress Disorder in France: Results From the WMH Survey. J Trauma Stress 28 (4):275-282. doi:10.1002/jts.22020

18. Hyland P, Vallières F, Cloitre M, Ben-Ezra M, Karatzias T, Olff M, Murphy J, Shevlin M (2021) Trauma, PTSD, and complex PTSD in the Republic of Ireland: prevalence, service use, comorbidity, and risk factors. Soc Psychiatry Psychiatr Epidemiol 56 (4):649-658. doi:10.1007/s00127-020-01912-x

19. Ishikawa H, Kawakami N, Kessler RC, World Mental Health Japan Survey C (2016) Lifetime and 12-month prevalence, severity and unmet need for treatment of common mental disorders in Japan: results from the final dataset of World Mental Health Japan Survey. Epidemiol Psychiatr Sci 25 (3):217-229. doi:10.1017/S2045796015000566

20. Ishikawa H, Tachimori H, Takeshima T, Umeda M, Miyamoto K, Shimoda H, Baba T, Kawakami N (2018) Prevalence, treatment, and the correlates of common mental disorders in the mid 2010's in Japan: The results of the world mental health Japan 2nd survey. J Affect Disord 241:554-562. doi:10.1016/j.jad.2018.08.050

21. Ito M, Takebayashi Y, Suzuki Y, Horikoshi M (2019) Posttraumatic stress disorder checklist for DSM-5: Psychometric properties in a Japanese population. J Affect Disord 247:11-19. doi:10.1016/j.jad.2018.12.086

22. Jacobi F, Hofler M, Strehle J, Mack S, Gerschler A, Scholl L, Busch MA, Hapke U, Maske U, Seiffert I, Gaebel W, Maier W, Wagner M, Zielasek J, Wittchen HU (2015) Twelve-months prevalence of mental disorders in the German Health Interview and Examination Survey for Adults - Mental Health Module (DEGS1-MH): a methodological addendum and correction. Int J Methods Psychiatr Res 24 (4):305-313. doi:10.1002/mpr.1479

23. Johns LE, Aiello AE, Cheng C, Galea S, Koenen KC, Uddin M (2012) Neighborhood social cohesion and posttraumatic stress disorder in a community-based sample: findings from the Detroit Neighborhood Health Study. Soc Psychiatry Psychiatr Epidemiol 47 (12):1899-1906. doi:10.1007/s00127-012-0506-9

24. Karatzias T, Hyland P, Bradley A, Cloitre M, Roberts NP, Bisson JI, Shevlin M (2019) Risk factors and comorbidity of ICD-11 PTSD and complex PTSD: Findings from a trauma-exposed population based sample of adults in the United Kingdom. Depress Anxiety 36 (9):887-894. doi:10.1002/da.22934

25. Karg RS, Bose J, Batts KR, Forman-Hoffman VL, Liao D, Hirsch E, Pemberton MR, Colpe LJ, Hedden SL (2012) Past Year Mental Disorders among Adults in the United States: Results from the 2008–2012 Mental Health Surveillance Study. In: CBHSQ Data Review. Substance Abuse and Mental Health Services Administration (US), Rockville (MD), pp 1-19

26. Kessler RC, Chiu WT, Demler O, Merikangas KR, Walters EE (2005) Prevalence, severity, and comorbidity of 12-month DSM-IV disorders in the National Comorbidity Survey Replication. Arch Gen Psychiatry 62 (6):617-627. doi:10.1001/archpsyc.62.6.617

27. Kim H, Lee J, Chang SM, Hong JP, Lee DW, Hahm BJ, Cho SJ, Park JI, Jeon HJ, Seong SJ, Park JE, Kim BS (2021) Prevalence of lifetime psychiatric disorders and suicidality in adults with subthreshold posttraumatic stress disorder: A population-based nationwide study in Korea. Psychol Trauma. doi:10.1037/tra0001185

28. Knipscheer J, Sleijpen M, Frank L, de Graaf R, Kleber R, Ten Have M, Dückers M (2020) Prevalence of Potentially Traumatic Events, Other Life Events and Subsequent Reactions Indicative for Posttraumatic Stress Disorder in the Netherlands: A General Population Study Based on the Trauma Screening Questionnaire. Int J Environ Res Public Health 17 (5). doi:10.3390/ijerph17051725

29. Koenen KC, Ratanatharathorn A, Ng L, McLaughlin KA, Bromet EJ, Stein DJ, Karam EG, Meron Ruscio A, Benjet C, Scott K, Atwoli L, Petukhova M, Lim CCW, Aguilar-Gaxiola S, Al-Hamzawi A, Alonso J, Bunting B, Ciutan M, de Girolamo G, Degenhardt L, Gureje O, Haro JM, Huang Y, Kawakami N, Lee S, Navarro-Mateu F, Pennell BE, Piazza M, Sampson N, Ten Have M, Torres Y, Viana MC, Williams D, Xavier M, Kessler RC (2017) Posttraumatic stress disorder in the World Mental Health Surveys. Psychol Med 47 (13):2260-2274. doi:10.1017/s0033291717000708

30. Lassemo E, Sandanger I, Nygård JF, Sørgaard KW (2017) The epidemiology of post-traumatic stress disorder in Norway: trauma characteristics and pre-existing psychiatric disorders. Soc Psychiatry Psychiatr Epidemiol 52 (1):11-19. doi:10.1007/s00127-016-1295-3

31. Leray E, Camara A, Drapier D, Riou F, Bougeant N, Pelissolo A, Lloyd KR, Bellamy V, Roelandt JL, Millet B (2011) Prevalence, characteristics and comorbidities of anxiety disorders in France: results from the "Mental Health in General Population" survey (MHGP). Eur Psychiatry 26 (6):339-345. doi:10.1016/j.eurpsy.2009.12.001

32. Levinson D, Zilber N, Lerner Y, Grinshpoon A, Levav I (2007) Prevalence of mood and anxiety disorders in the community: Results from the Israel National Health Survey. Isr J Psychiatry Relat Sci 44 (2):94-103

33. Lukaschek K, Kruse J, Emeny RT, Lacruz ME, von Eisenhart Rothe A, Ladwig KH (2013) Lifetime traumatic experiences and their impact on PTSD: a general population study. Soc Psychiatry Psychiatr Epidemiol 48 (4):525-532. doi:10.1007/s00127-012-0585-7

34. Maercker A, Forstmeier S, Wagner B, Glaesmer H, Brähler E (2008) [Post-traumatic stress disorder in Germany. Results of a nationwide epidemiological study]. Nervenarzt 79 (5):577-586. doi:10.1007/s00115-008-2467-5

35. Maercker A, Hecker T, Augsburger M, Kliem S (2018) ICD-11 Prevalence Rates of Posttraumatic Stress Disorder and Complex Posttraumatic Stress Disorder in a German Nationwide Sample. J Nerv Ment Dis 206 (4):270-276. doi:10.1097/nmd.0000000000000790

36. McManus S, Meltzer H, Brugha T, Bebbington P, Jenkins R (2009) Adult psychiatric morbidity in England, 2007: Results of a household survey. National Centre for Social Research

and the Department of Health Sciences, London, UK. doi:10.13140/2.1.1563.5205

37. Miller MW, Wolf EJ, Kilpatrick D, Resnick H, Marx BP, Holowka DW, Keane TM, Rosen RC, Friedman MJ (2013) The prevalence and latent structure of proposed DSM-5 posttraumatic stress disorder symptoms in U.S. national and veteran samples. Psychological Trauma: Theory, Research, Practice, and Policy 5 (6):501-512. doi:10.1037/a0029730

38. Muldoon OT, Downes C (2007) Social identification and post-traumatic stress symptoms in post-conflict Northern Ireland. Br J Psychiatry 191:146-149. doi:10.1192/bjp.bp.106.022038

39. Navarro-Mateu F, Tormo MJ, Salmeron D, Vilagut G, Navarro C, Ruiz-Merino G, Escamez T, Judez J, Martinez S, Kessler RC, Alonso J (2015) Prevalence of Mental Disorders in the South-East of Spain, One of the European Regions Most Affected by the Economic Crisis: The Cross-Sectional PEGASUS-Murcia Project. PLoS One 10 (9):e0137293. doi:10.1371/journal.pone.0137293

40. Ohayon MM, Shapiro CM (2000) Sleep disturbances and psychiatric disorders associated with posttraumatic stress disorder in the general population. Compr Psychiatry 41 (6):469-478. doi:10.1053/comp.2000.16568

41. Olaya B, Alonso J, Atwoli L, Kessler RC, Vilagut G, Haro JM (2015) Association between traumatic events and post-traumatic stress disorder: results from the ESEMeD-Spain study. Epidemiol Psychiatr Sci 24 (2):172-183. doi:10.1017/s2045796014000092

42. Parto JA, Evans MK, Zonderman AB (2011) Symptoms of posttraumatic stress disorder among urban residents. J Nerv Ment Dis 199 (7):436-439. doi:10.1097/NMD.0b013e3182214154

43. Sareen J, Cox BJ, Stein MB, Afifi TO, Fleet C, Asmundson GJ (2007) Physical and mental comorbidity, disability, and suicidal behavior associated with posttraumatic stress disorder in a large community sample. Psychosom Med 69 (3):242-248. doi:10.1097/PSY.0b013e31803146d8

44. Slade T, Johnston A, Oakley Browne MA, Andrews G, Whiteford HA (2009) 2007 National Survey of Mental Health and Wellbeing. methods and key findings. Aust N Z J Psychiatry 43 (7):594-605

45. Spitzer C, Barnow S, Völzke H, John U, Freyberger HJ, Grabe HJ (2009) Trauma, posttraumatic stress disorder, and physical illness: findings from the general population. Psychosom Med 71 (9):1012-1017. doi:10.1097/PSY.0b013e3181bc76b5

46. Van Ameringen M, Mancini C, Patterson B, Boyle MH (2008) Post-traumatic stress disorder in Canada. CNS Neurosci Ther 14 (3):171-181. doi:10.1111/j.1755-5949.2008.00049.x

47. van der Velden PG, Pijnappel B, van der Meulen E (2018) Potentially traumatic events have negative and positive effects on loneliness, depending on PTSD-symptom levels: evidence from a population-based prospective comparative study. Soc Psychiatry Psychiatr Epidemiol 53 (2):195-206. doi:10.1007/s00127-017-1476-8

48. Wells JE, Browne MA, Scott KM, McGee MA, Baxter J, Kokaua J, New Zealand Mental Health Survey Research T (2006) Prevalence, interference with life and severity of 12 month DSM-IV disorders in Te Rau Hinengaro: the New Zealand Mental Health Survey. Aust N Z J Psychiatry 40 (10):845-854. doi:10.1080/j.1440-1614.2006.01903.x

49. White J, Pearce J, Morrison S, Dunstan F, Bisson JI, Fone DL (2015) Risk of post-traumatic stress disorder following traumatic events in a community sample. Epidemiol Psychiatr Sci 24 (3):249-257. doi:10.1017/s2045796014000110

50. Yoo Y, Park HJ, Park S, Cho MJ, Cho SJ, Lee JY, Choi SH, Lee JY (2018) Interpersonal trauma moderates the relationship between personality factors and suicidality of individuals with posttraumatic stress disorder. PLoS One 13 (1):e0191198. doi:10.1371/journal.pone.0191198

51. Abeldaño RA, Fernández AR, Estario JC, Enders JE, Neira MJ (2014) [Screening for posttraumatic stress disorder in people affected by the 2010 earthquake in Chile.]. Cad Saude Publica 30 (11):2377-2386. doi:10.1590/0102-311x00141313

52. Acierno R, Ruggiero KJ, Galea S, Resnick HS, Koenen K, Roitzsch J, de Arellano M, Boyle J, Kilpatrick DG (2007) Psychological sequelae resulting from the 2004 Florida hurricanes: implications for postdisaster intervention. Am J Public Health 97 Suppl 1 (Suppl 1):S103-108. doi:10.2105/ajph.2006.087007

53. Agyapong VIO, Juhas M, Omege J, Denga E, Nwaka B, Akinjise I, Corbett SE, Brown M, Chue P, Li X-M, Greenshaw A (2019) Prevalence Rates and Correlates of Likely Post-Traumatic Stress Disorder in Residents of Fort McMurray 6 Months After a Wildfire. International Journal of Mental Health and Addiction 19 (3):632-650. doi:10.1007/s11469-019-00096-z

54. Alderman K, Turner LR, Tong S (2013) Assessment of the health impacts of the 2011 summer floods in Brisbane. Disaster Med Public Health Prep 7 (4):380-386. doi:10.1017/dmp.2013.42

55. Belleville G, Ouellet MC, Lebel J, Ghosh S, Morin CM, Bouchard S, Guay S, Bergeron N, Campbell T, MacMaster FP (2021) Psychological Symptoms Among Evacuees From the 2016 Fort McMurray Wildfires: A Population-Based Survey One Year Later. Front Public Health 9:655357. doi:10.3389/fpubh.2021.655357

56. Boscarino JA, Hoffman SN, Kirchner HL, Erlich PM, Adams RE, Figley CR, Solhkhah R (2013) Mental health outcomes at the Jersey Shore after Hurricane Sandy. Int J Emerg Ment Health 15 (3):147-158

57. Bryant RA, Waters E, Gibbs L, Gallagher HC, Pattison P, Lusher D, MacDougall C, Harms L, Block K, Snowdon E, Sinnott V, Ireton G, Richardson J, Forbes D (2014) Psychological outcomes following the Victorian Black Saturday bushfires. Aust N Z J Psychiatry 48 (7):634-643. doi:10.1177/0004867414534476

58. Carlsen HK, Gislason T, Benediktsdottir B, Kolbeinsson TB, Hauksdottir A, Thorsteinsson T, Briem H (2012) A survey of early health effects of the Eyjafjallajokull 2010 eruption in Iceland: a population-based study. BMJ Open 2 (2):e000343. doi:10.1136/bmjopen-2011-000343

59. Catapano F, Malafronte R, Lepre F, Cozzolino P, Arnone R, Lorenzo E, Tartaglia G, Starace F, Magliano L, Maj M (2001) Psychological consequences of the 1998 landslide in Sarno, Italy: a community study. Acta Psychiatr Scand 104 (6):438-442. doi:10.1034/j.1600-0447.2001.00512.x

60. Cerda M, Bordelois PM, Galea S, Norris F, Tracy M, Koenen KC (2013) The course of posttraumatic stress symptoms and functional impairment following a disaster: what is the lasting influence of acute versus ongoing traumatic events and stressors? Soc Psychiatry Psychiatr Epidemiol 48 (3):385-395. doi:10.1007/s00127-012-0560-3

61. Dell'osso L, Carmassi C, Stratta P, Massimetti G, Akiskal KK, Akiskal HS, Maremmani I, Rossi A (2012) Gender Differences in the Relationship between Maladaptive Behaviors and Post-Traumatic Stress Disorder. A Study on 900 L' Aquila 2009 Earthquake Survivors. Front Psychiatry 3:111. doi:10.3389/fpsyt.2012.00111

62. Di Fiorino M, Massimetti G, Corretti G, Paoli RA (2005) Post Traumatic Stress Psychopathology 8 years after a flooding in Italy. Bridg East West Psychiatry 3 (1):49-57

63. Fergusson DM, Horwood LJ, Boden JM, Mulder RT (2014) Impact of a major disaster on the mental health of a well-studied cohort. JAMA Psychiatry 71 (9):1025-1031. doi:10.1001/jamapsychiatry.2014.652

64. Flores AB, Collins TW, Grineski SE, Chakraborty J (2020) Disparities in Health Effects and Access to Health Care Among Houston Area Residents After Hurricane Harvey. Public Health Rep 135 (4):511-523. doi:10.1177/0033354920930133

65. French CE, Waite TD, Armstrong B, Rubin GJ, Beck CR, Oliver I (2019) Impact of repeat flooding on mental health and health-related quality of life: a cross-sectional analysis of the English National Study of Flooding and Health. BMJ Open 9 (11):e031562. doi:10.1136/bmjopen-2019-031562

66. Galea S, Brewin CR, Gruber MJ, Jones R, King DW, King LA, McNally RJ, Ursano RJ, Petukhova M, Kessler RC (2007) Exposure to Hurricane-related stressors and mental illness after Hurricane Katrina. Arch Gen Psychiatry 64 (12):1427-1434

67. Galea S, Tracy M, Norris F, Coffey SF (2008) Financial and social circumstances and the incidence and course of PTSD in Mississippi during the first two years after Hurricane Katrina. J Trauma Stress 21 (4):357-368. doi:10.1002/jts.20355

68. Gigantesco A, Mirante N, Granchelli C, Diodati G, Cofini V, Mancini C, Carbonelli A, Tarolla E, Minardi V, Salmaso S, D'Argenio P (2013) Psychopathological chronic sequelae of the 2009 earthquake in L'Aquila, Italy. J Affect Disord 148 (2-3):265-271. doi:10.1016/j.jad.2012.12.006

69. Gissurardóttir Ó S, Hlodversdóttir H, Thordardóttir EB, Pétursdóttir G, Hauksdóttir A (2019) Mental health effects following the eruption in Eyjafjallajökull volcano in Iceland: A population-based study. Scand J Public Health 47 (2):251-259. doi:10.1177/1403494817751327

70. Heir T, Piatigorsky A, Weisaeth L (2009) Longitudinal changes in recalled perceived life threat after a natural disaster. Br J Psychiatry 194 (6):510-514. doi:10.1192/bjp.bp.108.056580

71. Heo JH, Kim MH, Koh SB, Noh S, Park JH, Ahn JS, Park KC, Shin J, Min S (2008) A prospective study on changes in health status following flood disaster. Psychiatry Investig 5 (3):186-192. doi:10.4306/pi.2008.5.3.186

72. Hlodversdottir H, Petursdottir G, Carlsen HK, Gislason T, Hauksdottir A (2016) Long-term health effects of the Eyjafjallajökull volcanic eruption: a prospective cohort study in 2010 and 2013. BMJ Open 6 (9):e011444. doi:10.1136/bmjopen-2016-011444

73. Houston JB, Spialek ML, Stevens J, First J, Mieseler VL, Pfefferbaum B (2015) 2011 Joplin, Missouri Tornado Experience, Mental Health Reactions, and Service Utilization: Cross-Sectional Assessments at Approximately 6 Months and 2.5 Years Post-Event. PLoS Curr 7. doi:10.1371/currents.dis.18ca227647291525ce3415bec1406aa5

74. Kato H (1998) Posttraumatic symptoms among victims of the Great Hanshin-Awaji Earthquake in Japan. Psychiatr Clin Neurosci 52:S59-S65

75. Kukihara H, Yamawaki N, Uchiyama K, Arai S, Horikawa E (2014) Trauma, depression, and resilience of earthquake/tsunami/nuclear disaster survivors of Hirono, Fukushima, Japan. Psychiatry Clin Neurosci 68 (7):524-533. doi:10.1111/pcn.12159

76. Lowe SR, Sampson L, Gruebner O, Galea S (2015) Psychological resilience after Hurricane Sandy: the influence of individual- and community-level factors on mental health after a large-scale natural disaster. PLoS One 10 (5):e0125761. doi:10.1371/journal.pone.0125761

77. Marshall GNS, T. L; Elliott M. N.; rAYBURN; n: R.; Jaycox, L. H. (2007) Psychiatric disorders among adults seeking emergency disaster assistance after a wildland-urban interface fire. Psychiatric services

58 (4):509-514

78. Mason V, Andrews H, Upton D (2010) The psychological impact of exposure to floods. Psychol Health Med 15 (1):61-73. doi:10.1080/13548500903483478

79. Matthews V, Longman J, Bennett-Levy J, Braddon M, Passey M, Bailie RS, Berry HL (2020) Belonging and Inclusivity Make a Resilient Future for All: A Cross-Sectional Analysis of Post-Flood Social Capital in a Diverse Australian Rural Community. Int J Environ Res Public Health 17 (20). doi:10.3390/ijerph17207676

80. McLaughlin KA, Berglund P, Gruber MJ, Kessler RC, Sampson NA, Zaslavsky AM (2011) Recovery from PTSD following Hurricane Katrina. Depress Anxiety 28 (6):439-446. doi:10.1002/da.20790

81. Norris FH, Sherrieb K, Galea S (2010) Prevalence and consequences of disaster-related illness and injury from Hurricane Ike. Rehabil Psychol 55 (3):221-230. doi:10.1037/a0020195

82. Paranjothy S, Gallacher J, Amlot R, Rubin GJ, Page L, Baxter T, Wight J, Kirrage D, McNaught R, Palmer SR (2011) Psychosocial impact of the summer 2007 floods in England. BMC Public Health 11:145

83. Parslow RA, Jorm AF, Christensen H (2006) Associations of pre-trauma attributes and trauma exposure with screening positive for PTSD: analysis of a community-based study of 2,085 young adults. Psychol Med 36 (3):387-395. doi:10.1017/s0033291705006306

84. Priebe S, Grappasonni I, Mari M, Dewey M, Petrelli F, Costa A (2009) Posttraumatic stress disorder six months after an earthquake: findings from a community sample in a rural region in Italy. Soc Psychiatry Psychiatr Epidemiol 44 (5):393-397. doi:10.1007/s00127-008-0441-y

85. Priebe S, Marchi F, Bini L, Flego M, Costa A, Galeazzi G (2011) Mental disorders, psychological symptoms and quality of life 8 years after an earthquake: findings from a community sample in Italy. Soc Psychiatry Psychiatr Epidemiol 46 (7):615-621. doi:10.1007/s00127-010-0227-x

86. Ruggiero KJ, Gros K, McCauley JL, Resnick HS, Morgan M, Kilpatrick DG, Muzzy W, Acierno R (2012) Mental health outcomes among adults in Galveston and Chambers counties after Hurricane Ike. Disaster Med Public Health Prep 6 (1):26-32. doi:10.1001/dmp.2012.7

87. Shigemura J, Tanigawa T, Saito I, Numura S (2012) Psychological distress in workers at the Fukushima Nuclear Power Plant. J Am Med Assoc 308 (7):667-669. doi:10.1001/archinternmed.2012

88. Tsujiuchi T, Yamaguchi M, Masuda K, Tsuchida M, Inomata T, Kumano H, Kikuchi Y, Augusterfer EF, Mollica RF (2016) High Prevalence of Post-Traumatic Stress Symptoms in Relation to Social Factors in Affected Population One Year after the Fukushima Nuclear Disaster. PLoS One 11 (3):e0151807. doi:10.1371/journal.pone.0151807

89. Waite TD, Chaintarli K, Beck CR, Bone A, Amlot R, Kovats S, Reacher M, Armstrong B, Leonardi G, Rubin GJ, Oliver I (2017) The English national cohort study of flooding and health: cross-sectional analysis of mental health outcomes at year one. BMC Public Health 17 (1):129. doi:10.1186/s12889-016-4000-2

90. Yabe H, Suzuki Y, Mashiko H, Nakayama Y, Hisata M, Niwa S, Yasumura S, Yamashita S, Kamiya K, Abe M (2014) Psychological distress after the Great East Japan Earthquake and Fukushima Daiichi Nuclear Power Plant accident: results of a mental health and lifestyle survey through the Fukushima Health Management Survey in FY2011 and FY2012. Fukushima J Med Sci 60 (1):57-67. doi:10.5387/fms.2014-1

91. Adams RE, Boscarino JA (2005) Differences in mental health outcomes among Whites, African Americans, and Hispanics following a community disaster. Psychiatry 68 (3):250-265. doi:10.1521/psyc.2005.68.3.250

92. Besser A, Neria Y (2009) PTSD symptoms, satisfaction with life, and prejudicial attitudes toward the adversary among Israeli civilians exposed to ongoing missile attacks. J Trauma Stress 22 (4):268-275. doi:10.1002/jts.20420

93. Chipman KJ, Palmieri PA, Canetti D, Johnson RJ, Hobfoll SE (2011) Predictors of posttraumatic stress-related impairment in victims of terrorism and ongoing conflict in Israel. Anxiety Stress Coping 24 (3):255-271. doi:10.1080/10615806.2010.515304

94. DeLisi L, Maurizio A, Yost M, Papparozzi C, Katz C, Altesman J, Biel M, Lee J, Stevens P (2003) A Survey of New Yorkers After the Sept. 11, 2001, Terrorist Attacks. Am J Psychiatry 160 (4):780-783

95. DiGrande L, Neria Y, Brackbill RM, Pulliam P, Galea S (2011) Long-term posttraumatic stress symptoms among 3,271 civilian survivors of the September 11, 2001, terrorist attacks on the World Trade Center. Am J Epidemiol 173 (3):271-281. doi:10.1093/aje/kwq372

96. DiGrande L, Perrin MA, Thorpe LE, Thalji L, Murphy J, Wu D, Farfel M, Brackbill RM (2008) Posttraumatic stress symptoms, PTSD, and risk factors among lower Manhattan residents 2-3 years after the September 11, 2001 terrorist attacks. J Trauma Stress 21 (3):264-273. doi:10.1002/jts.20345

97. Elklit A (2007) Psychological consequences of a firework factory disaster in a local community. Soc Psychiatry Psychiatr Epidemiol 42 (8):664-668. doi:10.1007/s00127-007-0206-z

98. Fagan J, Galea S, Ahern J, Bonner S, Vlahov D (2003) Relationship of self-reported asthma severity and urgent health care utilization to psychological sequelae of the September 11, 2001 terrorist attacks on the World Trade Center among New York City area residents. Psychosom Med 65 (6):993-996. doi:10.1097/01.psy.0000097334.48556.5f

99. Farfel M, DiGrande L, Brackbill R, Prann A, Cone J, Friedman S, Walker DJ, Pezeshki G, Thomas P, Galea S, Williamson D, Frieden TR, Thorpe L (2008) An overview of 9/11 experiences and respiratory and mental health conditions among World Trade Center Health Registry enrollees. J Urban Health 85 (6):880-909. doi:10.1007/s11524-008-9317-4

100. Ferrando L, Galea S, Sainz Cortón E, Mingote C, García Camba E, Fernandez Líria A, Gabriel R (2011) Long-term psychopathology changes among the injured and members of the community after a massive terrorist attack. Eur Psychiatry 26 (8):513-517. doi:10.1016/j.eurpsy.2010.07.009

101. Galea S, Ahern J, Resnick H, Kilpatrick D, Bucuvalas M, Gold J, Vlahov D (2002) Psychological sequelae of the September 11 terrorist attacks in New York City. N Engl J Med 346 (13):982-987. doi:10.1056/NEJMsa013404

102. Genereux M, Maltais D, Petit G, Roy M (2019) Monitoring Adverse Psychosocial Outcomes One and Two Years After the Lac-Megantic Train Derailment Tragedy (Eastern Townships, Quebec, Canada). Prehosp Disaster Med 34 (3):251-259. doi:10.1017/S1049023X19004321

103. Hafstad GS, Thoresen S, Wentzel-Larsen T, Maercker A, Dyb G (2017) PTSD or not PTSD? Comparing the proposed ICD-11 and the DSM-5 PTSD criteria among young survivors of the 2011 Norway attacks and their parents. Psychol Med 47 (7):1283-1291. doi:10.1017/s0033291716002968

104. Hobfoll SE, Canetti-Nisim D, Johnson RJ, Palmieri PA, Varley JD, Galea S (2008) The association of exposure, risk, and resiliency factors with PTSD among Jews and Arabs exposed to repeated acts of terrorism in Israel. J Trauma Stress 21 (1):9-21. doi:10.1002/jts.20307

105. Jose R (2018) Mapping the Mental Health of Residents After the 2013 Boston Marathon Bombings. J Trauma Stress 31 (4):480-486. doi:10.1002/jts.22312

106. Miguel-Tobal JJ, Cano-Vindel A, Gonzalez-Ordi H, Iruarrizaga I, Rudenstine S, Vlahov D, Galea S (2006) PTSD and depression after the Madrid March 11 train bombings. J Trauma Stress 19 (1):69-80. doi:10.1002/jts.20091

107. Nandi A, Galea S, Ahern J, Vlahov D (2005) Probable Cigarette Dependence, PTSD, and Depression after an Urban Disaster: Results from a Population Survey of New York City Residents 4 Months after September 11, 2001. Psychiatry: Interpersonal and Biological Processes 68 (4):299-310

108. Rivière S, Schwoebel V, Lapierre-Duval K, Guinard A, Gardette V, Lang T (2008) Predictors of symptoms of post-traumatic stress disorder after the AZF chemical factory explosion on 21 September 2001, in Toulouse, France. J Epidemiol Community Health 62 (5):455-460. doi:10.1136/jech.2006.057273

109. Schlenger WE, Caddell JM, Ebert L, Jordan BK, Rourke KM, Wilson D, Thalji L, Dennis JM, Fairbank JA, Kulka RA (2002) Psychological reactions to terrorist attacks. Findings from the National Study of Americans' Reactions to September 11. JAMA 288 (5):581-588

110. Shalev AY, Tuval R, Frenkiel-Fishman S, Hadar H, Eth S (2006) Psychological responses to continuous terror: a study of two communities in Israel. Am J Psychiatry 163 (4):667-673. doi:10.1176/appi.ajp.163.4.667

111. Stein NR, Schorr Y, Krantz L, Dickstein BD, Solomon Z, Horesh D, Litz BT (2013) The differential impact of terrorism on two Israeli communities. Am J Orthopsychiatry 83 (4):528-535. doi:10.1111/ajop.12044

112. Tracy M, Hobfoll SE, Canetti-Nisim D, Galea S (2008) Predictors of depressive symptoms among israeli jews and arabs during the Al aqsa intifada: a population-based cohort study. Ann Epidemiol 18 (6):447-457. doi:10.1016/j.annepidem.2007.12.004
