# Supplementary material for: Work-related posttraumatic stress disorder in paramedics in comparison to data from the general population of working age. A systematic review and meta-analysis
Source: Front Public Health. 2023 Mar 9;11:1151248. doi: 10.3389/fpubh.2023.1151248 (PMC10035789; doi:10.3389/fpubh.2023.1151248)
Supplement: Supplementary file 1 [file Data_Sheet_1.ZIP › Supplementary_Material_PTSD_Paramedics_Frontiers.docx]

Supplementary Material

Work-related posttraumatic stress disorder in paramedics in comparison to data from the general population of working age. A systematic review and meta-analysis

Andreas Hoell*, Eirini Kourmpeli, Harald Dreßing

*** Correspondence:** Andreas Hoell: andreas.hoell@zi-mannheim.de

# Supplementary Material

**Supplement 1.** The Preferred Reporting Items of Systematic Reviews and Meta-Analyses (PRISMA) 2020 Checklist (consisting of 27 items) and corresponding sections within this meta-analysis.

**Supplement 2.** Search strings to identify post-traumatic stress disorder among paramedics for all included literature databases.

**Supplement 3.** Search strings for post-traumatic stress disorder in representative non-systematically trauma-exposed samples of high-income countries and in representative samples in high-income countries exposed to either Natural disasters or Human-made Disasters

**Supplement 4.** Methodological quality ratings of included studies of post-traumatic stress disorder among paramedics using the critical appraisal tool for cross-sectional studies (AXIS) tool.

**Supplement 5.** Characteristics of included studies on post-traumatic stress disorder in paramedics.

**Supplement 6.** Characteristics of included studies on post-traumatic stress disorder in representative non-systematically trauma-exposed samples, and representative trauma-exposed samples of high-income countries.

**Supplement 7.** Funnel plot of included studies on post-traumatic stress disorder in paramedics.

**Supplement 8.** Forest plot of 12-month prevalence of post-traumatic stress disorder in paramedics.

**Supplement 9.** Forest plot of 12-month prevalence of post-traumatic stress disorder among the general population not systematically exposed to traumatic events.

**Supplement 10.** Forest plot of 12-month prevalence of post-traumatic stress disorder among the general population exposed to human-made disasters.

**Supplement 11.** Forest plot of 12-month prevalence of post-traumatic stress disorder among the general population exposed to natural disasters.

**Supplement 12.** Funnel plot of included studies on post-traumatic stress disorder in representative non-systematically trauma-exposed samples of high-income countries.

**Supplement 13.** Funnel plot of included studies on representative samples of high-income countries exposed to human-made disasters.

**Supplement 14.** Funnel plot of included studies on representative samples of high-income countries exposed to natural disasters.
