# Supplementary figures and images for: Work-related posttraumatic stress disorder in paramedics in comparison to data from the general population of working age. A systematic review and meta-analysis
Source: Front Public Health. 2023 Mar 9;11:1151248. doi: 10.3389/fpubh.2023.1151248 (PMC10035789; doi:10.3389/fpubh.2023.1151248)

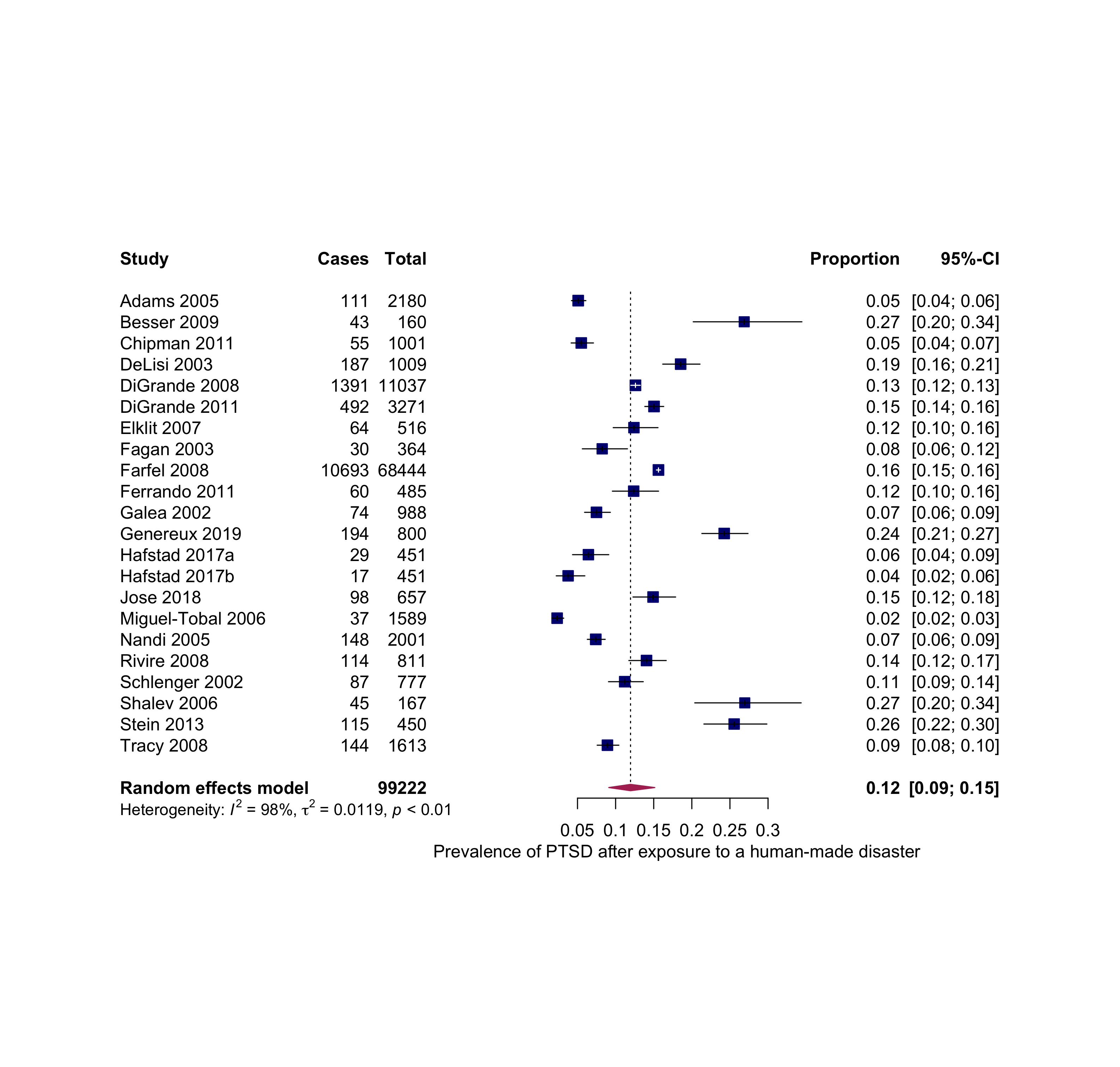

Supplement: Supplementary file 1 [file Data_Sheet_1.ZIP › S10 Forest_plot_general_pop_HMD.png]

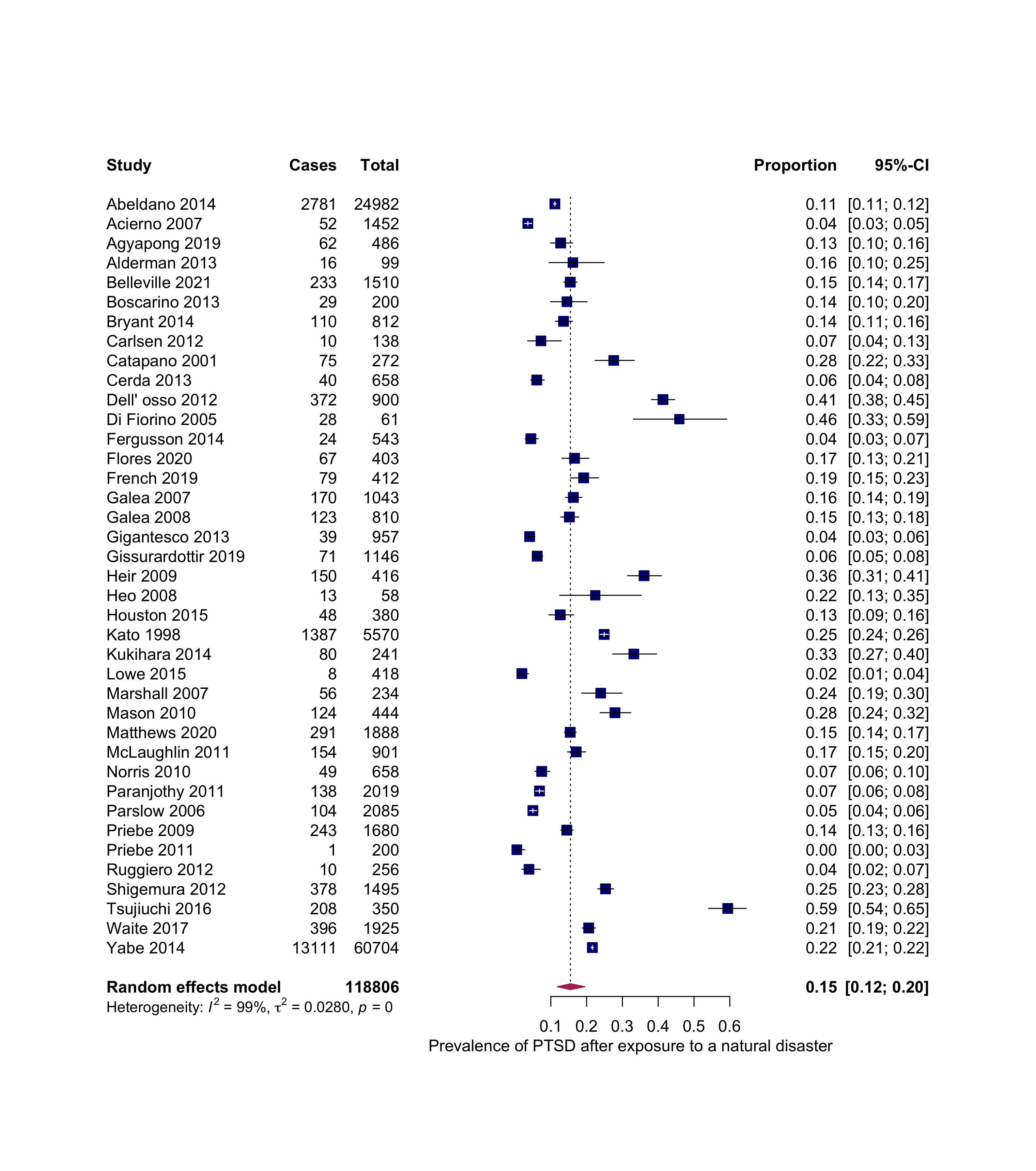

Supplement: Supplementary file 1 [file Data_Sheet_1.ZIP › S11_Forest_plot_general_pop_ND.png]

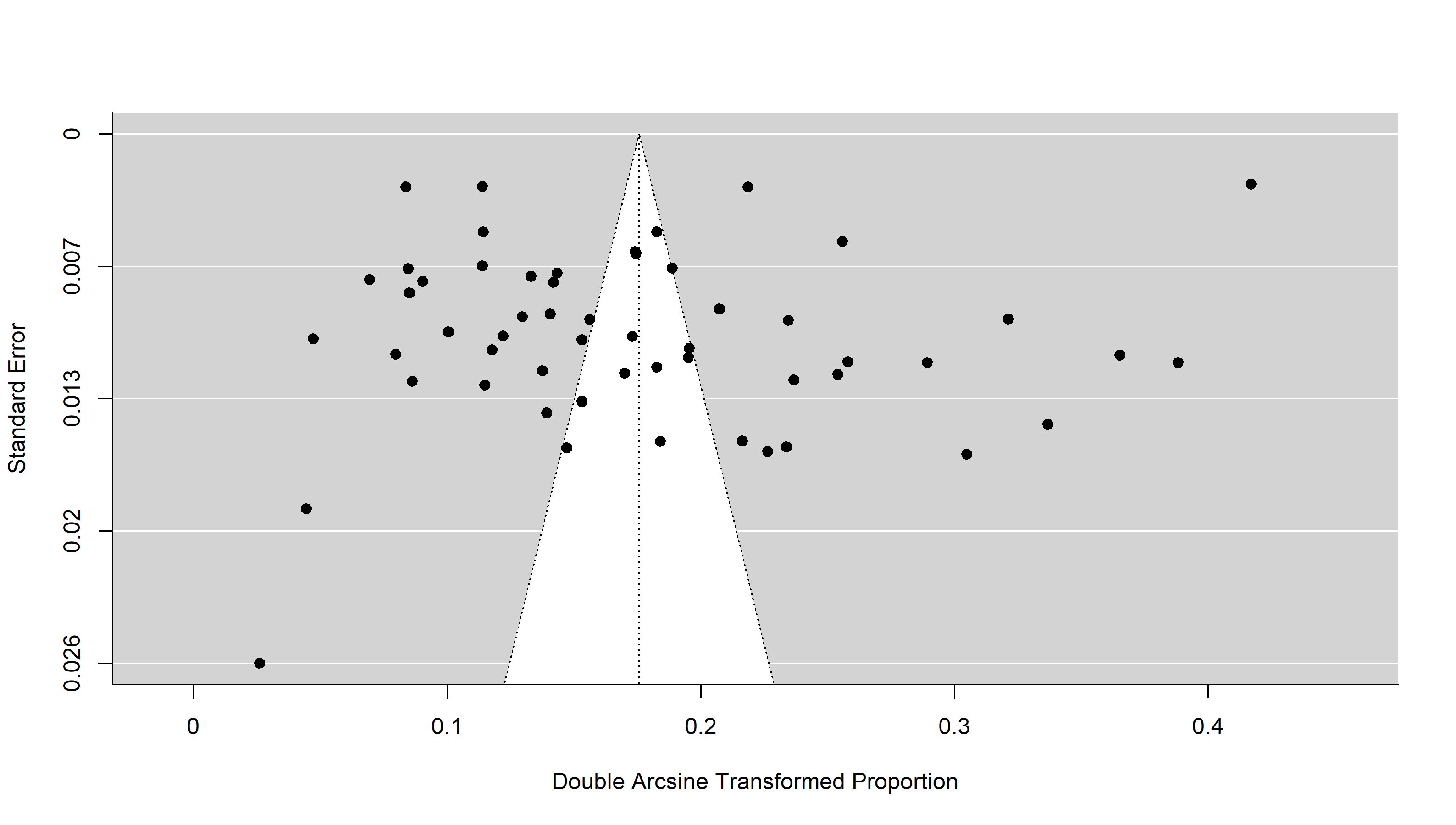

Supplement: Supplementary file 1 [file Data_Sheet_1.ZIP › S12 Funnelplot_general_pop_UX.png]

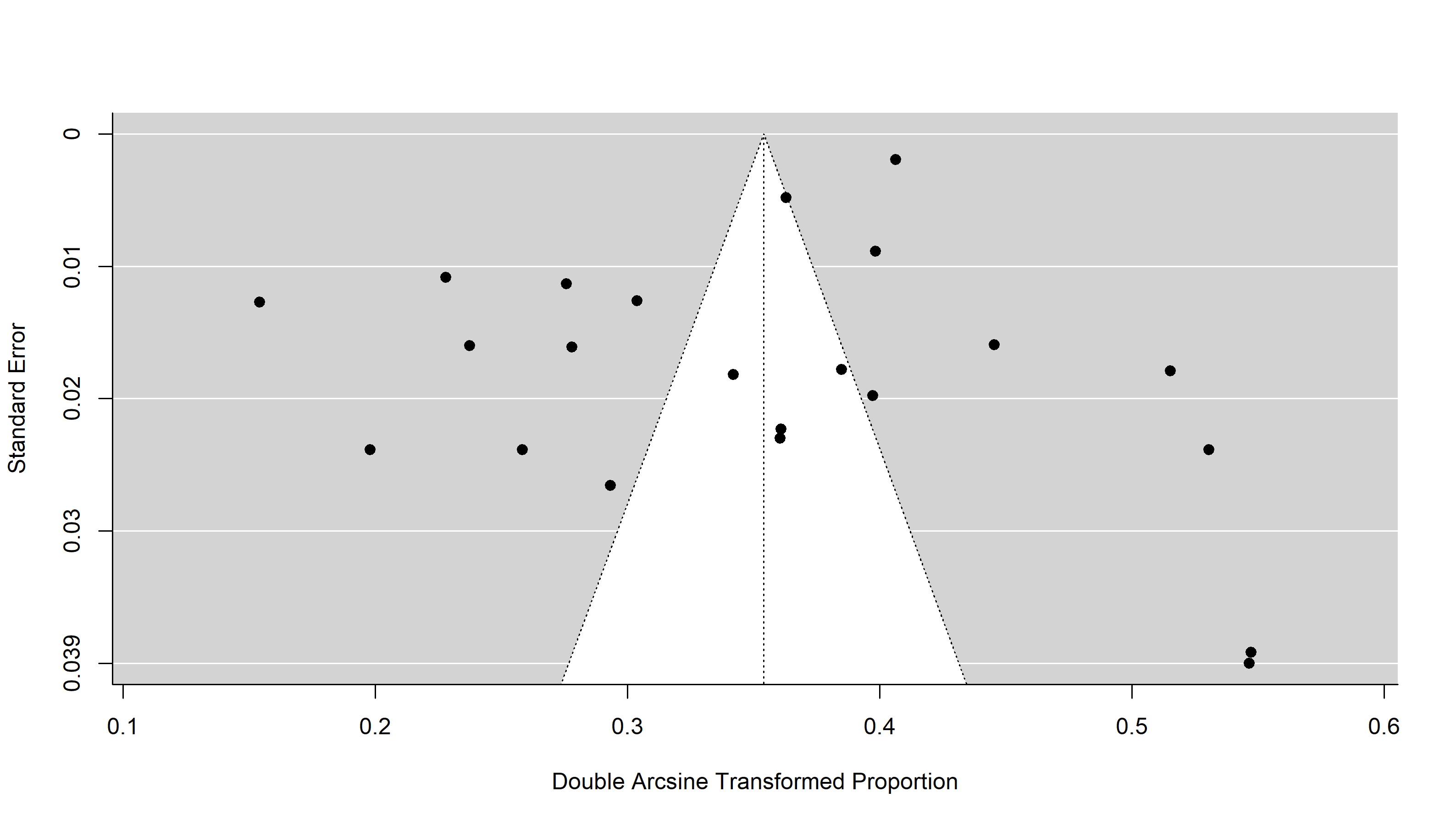

Supplement: Supplementary file 1 [file Data_Sheet_1.ZIP › S13 Funnelplot_general_pop_HMD.png]

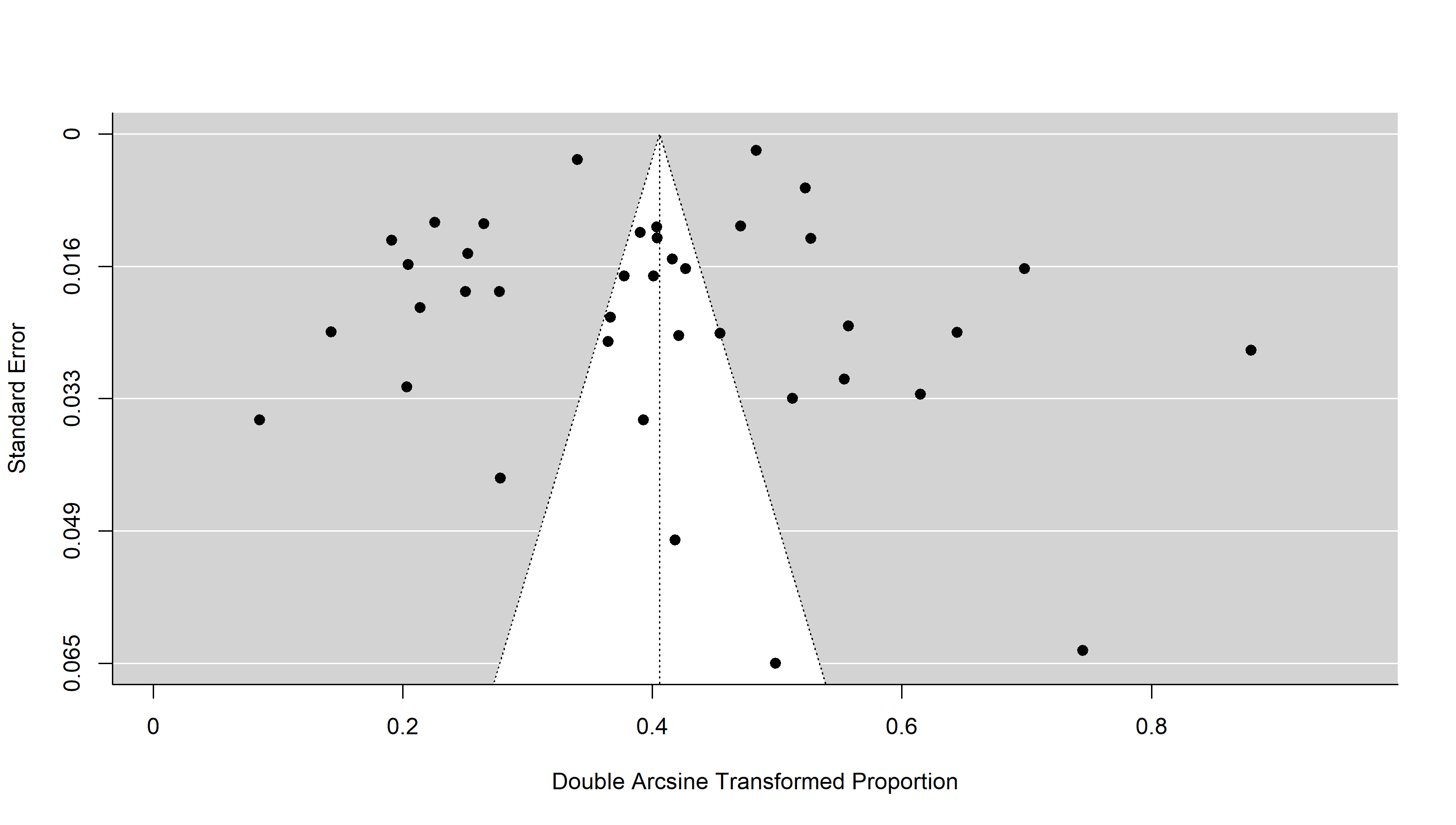

Supplement: Supplementary file 1 [file Data_Sheet_1.ZIP › S14 Funnelplot_general_pop_ND.png]

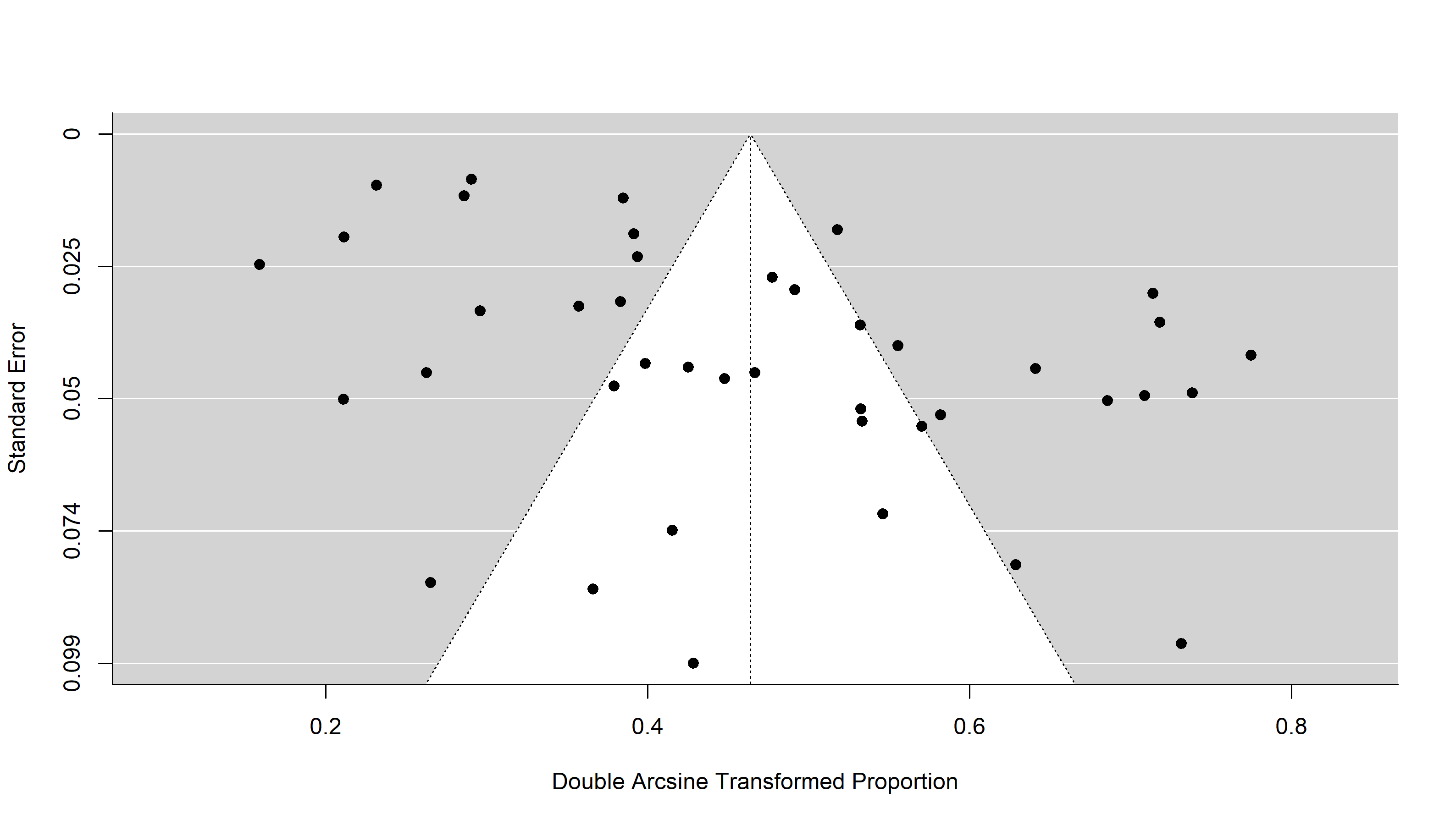

Supplement: Supplementary file 1 [file Data_Sheet_1.ZIP › S7 Funnelplot_Paramed.png]

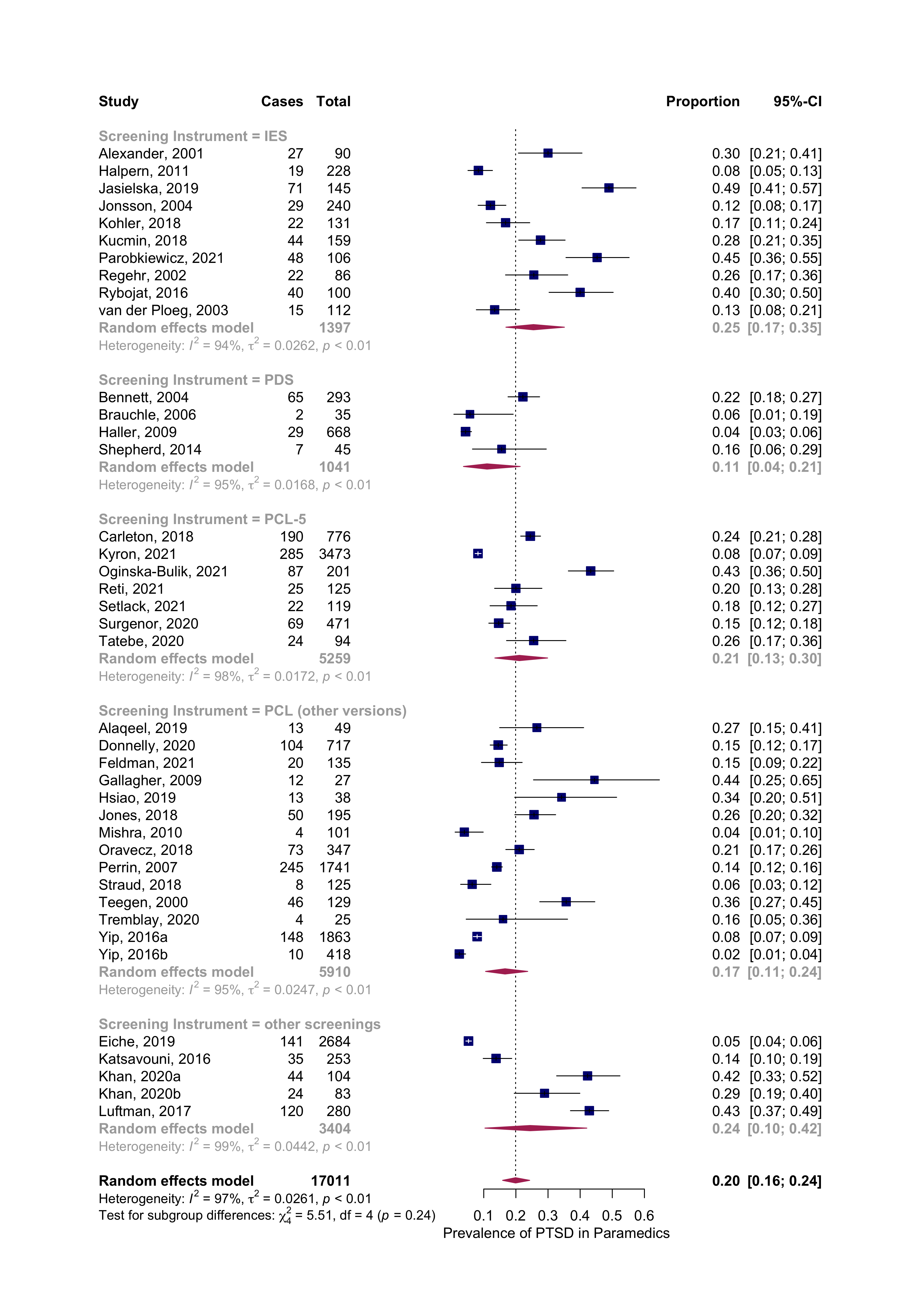

Supplement: Supplementary file 1 [file Data_Sheet_1.ZIP › S8 Forest_plot_Paramedics_Screenings.png]

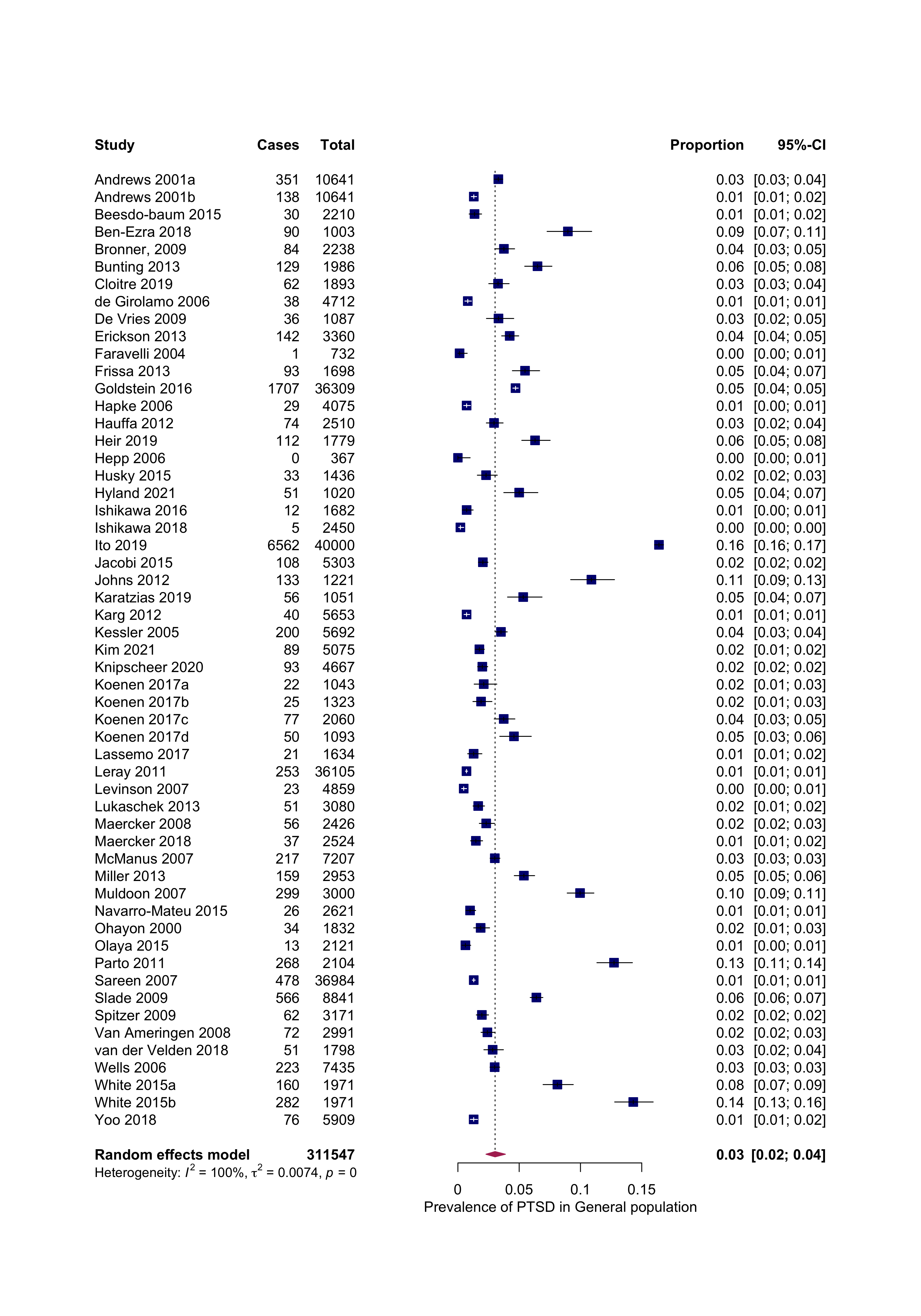

Supplement: Supplementary file 1 [file Data_Sheet_1.ZIP › S9 Forest_plot_general_pop_UX.png]
